# The emergence of sequence-dependent structural motifs in stretched, torsionally constrained DNA

**DOI:** 10.1093/nar/gkz1227

**Published:** 2020-01-13

**Authors:** Jack W Shepherd, Robert J Greenall, Matt I J Probert, Agnes Noy, Mark C Leake

**Affiliations:** 1 Department of Physics, University of York, York YO10 5DD, UK; 2 Department of Biology, University of York, York,YO10 5NG, UK

## Abstract

The double-helical structure of DNA results from canonical base pairing and stacking interactions. However, variations from steady-state conformations resulting from mechanical perturbations in cells have physiological relevance but their dependence on sequence remains unclear. Here, we use molecular dynamics simulations showing sequence differences result in markedly different structural motifs upon physiological twisting and stretching. We simulate overextension on different sequences of DNA ((AA)_12_, (AT)_12_, (CC)_12_ and (CG)_12_) with supercoiling densities at 200 and 50 mM salt concentrations. We find that DNA denatures in the majority of stretching simulations, surprisingly including those with over-twisted DNA. GC-rich sequences are observed to be more stable than AT-rich ones, with the specific response dependent on the base pair order. Furthermore, we find that (AT)_12_ forms stable periodic structures with non-canonical hydrogen bonds in some regions and non-canonical stacking in others, whereas (CG)_12_ forms a stacking motif of four base pairs independent of supercoiling density. Our results demonstrate that 20–30% DNA extension is sufficient for breaking B-DNA around and significantly above cellular supercoiling, and that the DNA sequence is crucial for understanding structural changes under mechanical stress. Our findings have important implications for the activities of protein machinery interacting with DNA in all cells.

## INTRODUCTION

In the cell, DNA is constantly under mechanical perturbation from a range of proteins as well as protein/nucleic acid based molecular machines. These perturbations are necessary for a wide range of DNA functions, including replication, repair, gene expression, and chromosomal packaging (1,2). Single-molecule force spectroscopy experiments have shown that DNA can be over-stretched from 10% up to 70% beyond its relaxed contour length at a nearly constant force of around 60–70 pN (3,4). The observed force plateau has been associated with a conformational transition from the canonical B-DNA to the extended S-DNA (2), in which the base pairs are highly inclined with respect to the helical axis, and to DNA melting (5,6). The B–S transition has been observed to be dependent on a number of different factors including supercoiling density (7), AT/GC content (8), and salt concentration (5,6,9). In particular, it was observed that AT-rich DNA, in particular poly d(A–T)·poly d(A–T) fragments, favoured the extension to the S-form in contrast to GC-rich or poly d(C–G)·poly d(C–G) (8). The effect of torsional stress is dependent on whether the supercoiling applied is positive or negative: unwinding greatly reduces the force at which the B–S transition occurs while over-winding increases the overstretch needed for the transition (7). For significantly over-twisted DNA (with a supercoiling density σ > 0.037, where σ =$\frac{{{\rm Lk} - {{\rm Lk}_0}}}{{{{\rm Lk}_0}}}$ and Lk and Lk_0_ are the number of helical turns in the DNA and its natural number of helical turns, respectively), stretching DNA using magnetic tweezers showed the emergence of a Pauling-like DNA (P-DNA) structure, in which the bases were flipped outside and the two helical backbones were on the inside with drastically reduced helical pitch (10). Meanwhile under applied torque without stretch, DNA demonstrates a range of conformations which are sequence-specific and co-operative, including left-handed DNA (11). Finally, the effect of salt has been investigated with optical tweezers and dyes which can bind both single-stranded and double-stranded DNA with different fluorescence emission spectra (6). It was found that in general a higher salt concentration (200 mM) stabilized the duplex and resulted in the emergence of fewer melting bubbles, although this work was done in the absence of torsional constraints and it is therefore unresolved whether the mechanical perturbation would dominate over the ionic strength.in the case of a torsionally constrained and stretched molecule

It is difficult to visualize experimentally the effect of stretching and twisting DNA at the single base pair level. Using the most advanced dynamic super-resolution fluorescence microscopy methods for DNA, the spatial localization precision for pinpointing individual reporter dye molecules is at best a few tens of nm (12–14). This level of precision is equivalent to the spatial separation of around 100 bp and so precludes the extraction of information about the orientation or specific interactions of any given nucleotide. As a result, structural transitions have been inferred indirectly through considerations of the effects of force and torque while the structures themselves have been found using techniques such as space-filling algorithms (10,15). By contrast, computer simulations are able to provide atomically precise predictions for different overstretched DNA conformations and emergent structural motifs. These detailed descriptions may also be correlated with experimental data to give a more holistic understanding of the DNA structural dynamics.

Initial investigations of S-DNA using atomic molecular dynamics (MD) described a ladder-like structure where the Watson–Crick (WC) hydrogen bonds were mostly maintained. Base–base intra-strand stacking was preserved through a high inclination of the base pairs with respect to the molecular axis or was substituted by stacking between bases from opposite strands (i.e. inter-strand) (16–18). Later, it was also demonstrated that the structure of over-stretched DNA consists of a range of conformations in which the S-DNA motif can co-exist both with other motifs and short melting bubbles, especially in AT-rich segments (7,19–24). Further computer simulations described in atomic detail the P-form of over-stretched and over-twisted DNA (25). A combined experiment/steered molecular dynamics study (26) showed that under extreme over-stretching (with forces around 1 nN), a force plateau is observed for torsionally constrained DNA but not for unconstrained, confirming that removing the torsional freedom forces overstretched DNA into novel conformations, and specifically produces and an underwound P-DNA-like structure. Structures taken from these simulations showed DNA forming a bases-out structure in coexistence with zip-like DNA, in which bases from opposing strands interlock alternately (27,28). However, the details of the transition pathway in the early stages of overstretching and torsion- and sequence-dependence were not evaluated. Additionally, a combined quantum mechanics/molecular mechanics (QM/MM) study of sequence-specific melting pathways demonstrated that the AMBER forcefields agree with QM calculations and therefore that the melting pathways observed are more likely to be reliable (29). However, these simulations were undertaken without torsional restraints on the DNA.

Simulations of the effect of torque on linear DNA structures has been comparatively underexplored. Simulations in which torsional constraints were applied have shown that underwound DNA has a greater predisposition than relaxed DNA to form melting bubbles, and that this propensity is strongly dependent on sequence: denaturation was observed in an (AT)_3_ segment in linear DNA (30) and prominently in AT-rich areas in under-twisted small DNA circles that were between 60 and 110 bp in length (31). An infinite linear tract of DNA when under-twisted showed DNA partitioning its perturbations such that regions of extreme disruption coexist with regions that remain relatively close to the canonical B-form DNA structure, and that this phenomenon is sequence-dependent (32). The appearance of twist-based disruptions is not only due to the sequence directly at the perturbation: an imposed supercoiling density on an otherwise unconstrained fragment of DNA showed that pyrimidine-purine and, to a lesser extent, purine-purine steps were able to absorb significant twist, depending on the base pair sequence to either side (33).

Although atomic simulations have the capacity for generating highly resolved structural details, they are computationally very demanding and so the trajectory length is usually relatively short. Coarse-grained models like oxDNA (34) and 3SPN (35), which describe a DNA molecule with a significantly reduced number of degrees of freedom, are an alternative, powerful tool for describing the mechanism of DNA stretching over longer length and time scales at the cost of spatial precision (36,37). Force-induced melting was initially described by oxDNA (36), and later the coexistence of denaturation bubbles and S-DNA was observed using a 3SPN-based potential (37).

Despite the sampling advantages of coarse-grained methods, the lack of degrees of freedom means that some models, including oxDNA (36), do not capture the physics of non-canonical interactions and therefore cannot reproduce all conformations found experimentally or through all-atom simulations. Atomistic MD is therefore the most structurally detailed method of assessing base pair level DNA structure under significant mechanical perturbation, although care must be taken to ensure that conformational space has been properly sampled as experimental time scales are in general not accessible. However, the combined effects of precisely imposed physiological-range stretching and twisting perturbations together with sequence dependence has not been studied in depth with simulation to date. In our present work, we report a comprehensive set of atomically precise molecular dynamics simulations to investigate the structural impact of DNA over-extension on topologically-constrained molecules within the range of normal supercoiling density observed *in vivo* (which here we bound by σ = ±0.068) (38,39). We use four different sequences (poly d(A)·poly d(T), poly d(A–T)·poly d(A–T), poly d(C)·poly d(G) and poly d(C–G)·poly d(C–G)) which cover a significant variety of known DNA flexibility (40), as well as two biologically important motifs: the TATA box (41), which is used for transcription factor binding, and the CGCG box, which is known to be part of many signalling pathways in plants (42) and which can occur within CpG islands, regions of high CG density which are found in approximately 40% of mammalian gene promoter regions (43).

We focus on the early-stage overstretching regime (just up to the level of *ca*. 40% beyond the relaxed contour length) as this is the known upper-limit of mechanical distortion that can be achieved through protein-binding (for example, in the TATA-binding protein (44), and the recombinase enzyme RecA (45)) and thus enables us to explore the most physiologically relevant regime. We find that this level of over-stretching is sufficient to disrupt the B-DNA form in spite of the different torsional constraints, and that melting bubble behaviour and non-canonical structure formation were found to be significantly dependent on sequence. In contrast to previous work, we find that stretching torsionally constrained DNA leads to melting events earlier in the over-stretching regime because the propensity of DNA to overwind when stretched (46) is not available as is largely true in physiological situations. We further examine two salt concentration regimes – one at a physiological level of 200 mM and one low-salt condition at 50 mM, in order to understand the extent to which mechanical perturbation dominates over local conditions.

## MATERIALS AND METHODS

### Software simulation platforms

All simulations were set up with the AMBER 17 suite of programs and performed using the CUDA implementation of AMBER’s pmemd program (47). The initial 24 bp structures for four different linear DNA fragments were obtained using AMBER’s NAB utility (47) for the following sequences: (AA)_12_, (AT)_12_, (CC)_12_ and (CG)_12_ for exploring the effects of sequence dependence. Note that sequences did not have GC caps at each end because terminal base pairs were under constraints in all simulations so could not suffer from helix-end melting. This setup mimics force-extension experiments with both ends fixed to a *ca*. micron diameter bead or a surface preventing unpeeling (6), in contrast to many other experiments of this kind with just one attached strand (5). The molecular forcefield used for the DNA was the AMBER Parm99 forcefield (48) with bsc0 (49) and bsc1 (50) corrections for backbone dihedrals.

### Simulations in implicit solvent

The Generalized Born (GB) model (51,52) was applied to define the solvent implicitly, together with an effectively infinite long-range electrostatic cut-off, the latest GBneck2 corrections and mbondi3 Born radii set for a better reproduction of molecular surfaces, salt bridges and solvation forces (53). Systems were minimized using a combination of steepest descent and conjugate gradient methods. Simulations were run at constant temperature (300 K), which was maintained by the Langevin thermostat (54), using an effective salt concentration of either 200 or 50 mM determined by the Debye−Hückel screening parameter. Integration time steps were set at 1 fs and the DNA structure was written to disk every 1 ps.

**Figure 1.**
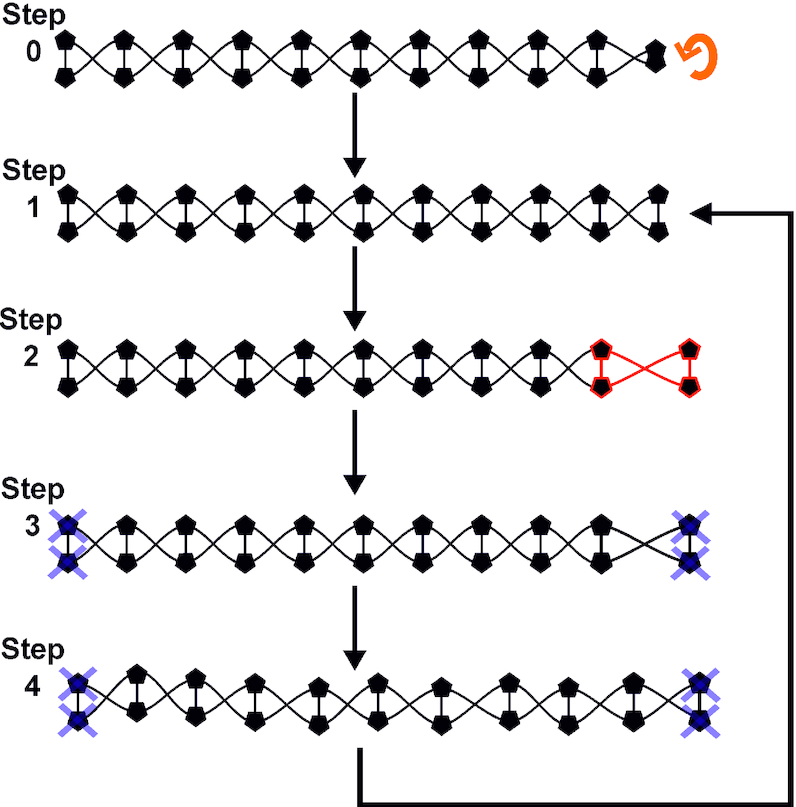
Schematic of stretching protocol. Step 0: A terminal base pair of a fragment is rotated with a 2D rotation matrix such that in step 1 an initial structure is obtained. Step 2: a terminal base pair (highlighted red) is displaced from its starting position by 1 Å. Step 3; the structure is minimized with harmonic constraints at both ends (grey crosses). Step 4: the system is subjected to a production run of 0.5 ns and the final frame is used as the input for the next round of stretching, minimizing and modelling.

Before the start of the simulation, the DNA was put into an appropriately supercoiled state. Because the helical axis was parallel to the z axis, this was done with an ordinary 2D rotation matrix applied to one of the terminal base pairs (step 0 in Figure 1). To stretch DNA while maintaining torsional constraints, fixed harmonic traps with an effectively infinite associated force constant of 500 kcal/mol/Å^2^ were applied to all atoms in the terminal base pairs (see Figure 1). DNA was stretched on a series of umbrella sampling trajectories, where one of the terminal base pairs was moved 1 Å from the centre of the molecule along the helical axis (Figure 1). The final frame of the previous umbrella sampling window was used as the starting structure for the next stretching event (Figure 1). Through analysis of temperatures, potential energies, and RMSd deviation from the starting structure, the system was found to equilibrate rapidly, both energetically and structurally, after the perturbation (see Supplementary Figure S1) as the increment on each extension step falls within the range of end-to-end thermal fluctuations found in a piece of unconstrained DNA (55). Every simulation window was 500 ps in width, using an overall stretch rate of 2 Å/ns. On a single umbrella sampling trajectory, each DNA molecule was extended by a total of 30 Å, resulting in an increase in contour length of ∼39.6% and a total run time of 15 ns. Each of the four DNA sequences considered was modelled using nine different supercoiling densities spanning a range used in previous magnetic tweezers studies (σ values of: 0, ±0.017, ±0.034, ±0.051 and ±0.068), whose negative extremity is also comparable to that estimated for live bacteria (39); the four simulations for the physiologically most relevant supercoiling density of σ = −0.068 are shown in Supplementary Movies 1–4. All sequences at all torsions were performed in both 50 and 200 mM salt concentrations, leading to 1080 ns of simulation time in total in implicit solvation.

### Trajectory analysis

Trajectories were analysed using cpptraj (56) to find canonical and non-canonical hydrogen bonds using distance and angle cut-offs of 3.5 Å and 120°, respectively. The esander routine was used to extract van der Waals (vdW) interactions between successive nucleotides to find canonical stacking interactions, and between a nucleotide and every non-neighbour nucleotide for the non-canonical stacking energies. Interaction energy calculations here were all performed in implicit solvation with the same forcefield and salt conditions as were used during the simulation. For each base pair, hydrogen bonds and stacking energies were averaged for each stretch extent in each simulation and these values were used to describe the structural details of DNA stretching and to quantify the presence of melting bubbles (defined to be two or more sequential base pairs which have on average less than one canonical WC hydrogen bond present). Representative structures were then extracted using VMD (57) or Chimera (58). Two base pairs at each end of the fragment were not considered in the trajectory analysis to avoid end-effects which in this structure are likely to produce interfaces between constrained Watson-Crick style base pairing and the structurally plastic central region. We are careful also to draw our example structures from the centre of the fragment, i.e. the central 14 bp, and in the heatmaps presented in the Results section the horizontal axis runs from bp 3 to 22.

We calculated the total force on every atom and for each trajectory in the absence of harmonic restraints using the post-processing AMBER’s sander utility. Of these, the nine backbone atoms of the terminal base pairs were selected and used to calculate the mean average end-to-end force for each given simulation frame. The global mean average end-to-end force was then calculated from these data across all *ca*. 15 000 uncorrelated individual simulation frames to produce a single uncorrected mean average force estimate that was experienced for each sequence for each whole simulation. Although, in principle, it would in general be desirable to determine a force-extension curve by plotting the force at each time step, thermal noise precluded this. In order to control for thermal noise, we took a control simulation which was run under identical conditions to the stretching simulation but in the absence of any end restraints, and found the mean average end-to-end force in an identical way. This we then subtracted from the mean force calculated for the pulling simulation in order to isolate the effect of pulling from thermally-induced forces. Using this method we obtained corrected mean average force estimates, *F*_avg_, between 8 and 33 pN for the different sequences. Specifically, for the maximally under-twisted simulations we obtained: (i) (AA)_12_*F*_avg_ = 8 pN, (ii) (AT)_12_*F*_avg_ = 27 pN, (iii) (CC)_12_*F*_avg_ = 33 pN and (iv) (CG)_12_*F*_avg_ = 25 pN, with an associated standard error in the mean (SEM) for each of ∼3 pN. Note, the errors were essentially driven by thermal noise therefore, over a sufficiently long time scale, as for our ∼15 000 simulation frames, we expect this to result in the same approximate uncertainty in the forces for each identical (except for sequence) simulation, thus resulting in similar SEM values of the ∼3 pN we estimate here throughout. This relatively large range of forces is reflective of the stochastics inherent in the data; it is worth noting that for an optical or magnetic tweezers experiment the integration time of a single data point would likely be in excess of the time scale of our entire implicit solvent simulation, thus smoothing the noise in a way that is not accessible for us here. However, that range of forces is at the high end of the physiological range and in line with previous tweezers experiments on single DNA molecules: it indicates, broadly, that we are not applying an excessive mechanical perturbation to the DNA compared to either native cellular conditions or to previous experimental studies.

### Simulations in explicit solvent

To ensure reasonable accuracy, a full stretching simulation in explicit solvent was undertaken for (AT)_12_ at σ = −0.068 and (CC)_12_ at ±0.068. The procedure for stretching was as indicated above, with the three simulations shown in Supplementary Movies 5–7. The integration time was 0.5 fs and at each level of overstretch the system was simulated for 1 ns, giving a total trajectory length of 30 ns. Structures were written to disk every 0.5 ps, resulting in 2000 frame long trajectories. The structure were solvated in TIP3P water (59) and 200 mM NaCl (60,61). The electrostatic cut-off was set at 1.2 nm, the NPT ensemble was used, with the Langevin thermostat (54) and Berendsen barostat (62). Covalent bonds with hydrogens were constrained using the SHAKE method (63). Analysis subsequently was carried out as for the implicit solvent simulations.

## RESULTS

### Overview of results

In this work, three physical parameters (ionic strength, supercoiling density, and imposed overstretch) are varied and the structural response of DNA is assessed. In general, we have found that explicit solvation models are comparable to the equivalent implicit solvation simulations under the physically constrained conditions we have simulated. Further, we have found that the supercoiling density only weakly modifies the behaviour. Instead, the response of the DNA to torsionally constrained over-stretching is primarily dependent on sequence. We find that in all cases AT-rich DNA is significantly less stable than CG-rich fragments. However, in the purine/pyramidine repeating dinucleotide sequences (i.e. (AT)_12_ and (CG)_12_) we find that stable structural motifs are formed. In the case of (AT)_12_ this takes the form of a dual motif in which small individual regions are stabilized either by the formation of non-canonical stacking interactions or by the formation of stable non-canonical hydrogen bonds. Most strikingly, in the (CG)_12_ sequence we see a repeating 4 bp motif characterized by a well-defined shift in canonical and non-canonical stacking energies, the loss of two canonical hydrogen bonds and the stable formation of one non-canonical hydrogen bond. Meanwhile, the (CC)_12_ sequence remains almost entirely in a canonical conformation, while the (AA)_12_ melts at the beginning of the simulations. These different sequence-dependent behaviours are summarized in Table 1.

**Table 1.** Summary of observed motifs under overstretching for the four sequences investigated

Sequence repeating unit	Response under over-stretching	Notes
AA	Melts early in simulation	True across ionic strengths and supercoiling densities
AT	Forms dual-mode motifs	Present in both ionic strengths and in explicit solvation model
CC	Assumes a highly inclined but canonical structure	Most stable sequence; similar behaviour in explicit solvent with the caveat that in explicit solvent small melting bubble formation is seen for both over and under-twisted DNA rather than just under-twisted as in implicit solvent
CG	Forms characteristic 4 bp motif	Extremely stable motif; formed irrespective of salt or supercoiling. It appears earlier (∼15% of extension) on under-twisted DNA in comparison with over-twisted DNA (∼30% of extension)

In the following subsections the results will be discussed in more detail. Firstly the explicit solvent simulations will be presented and the similarity to implicit solvent calculations examined. Then, the results of the implicit solvent simulations at 200 mM salt and with σ = −0.068 are shown. The complementary σ = 0.068 and 50 mM salt simulations are also discussed here, with reference to the full figures in Supplementary Information.

### Explicit solvation results are in reasonable agreement with implicit solvent simulations

**Figure 2.**
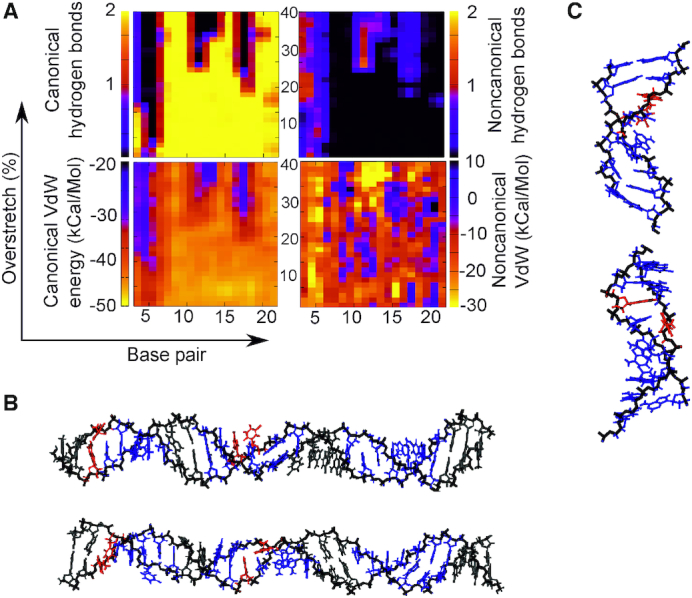
(AT)12 with σ = −0.068 simulated in 200 mM salt with explicit (TIP3P) solvation and stretched by 40% in increments. Here, the umbrella sampling windows are 1 ns long. We find that the stability of the DNA duplex is increased by the explicit solvent with significant regions retaining B-DNA like interactions. However, in disrupted regions the behaviour is broadly similar to that in Figure 6, and presents similar interlinked structures in panel B. Here red indicates non-canonical hydrogen bonding >1/bp and blue indicates non-canonical stacking <–10 kcal/mol.

Three explicit solvent simulations were undertaken in 200 mM NaCl as specified in the Methods section. Specifically, we simulated (AT)_12_ with σ = −0.068, and (CC)_12_ with σ = ±0.068. As can be seen in Figure 2, the (AT)_12_ simulation produced results comparable to the implicit solvent case in Figure 6, though the magnitude of the effect has been diminished. We see the characteristic loss of canonical hydrogen bonds and stacking interactions, with associated increase in both non-canonical stacking and hydrogen bonding in those areas. This is similar to the changes already observed in the implicitly solvated simulations, despite these coarser models not capturing explicit solvent-solute interactions. We observe a slight offset between the non-canonical stacking and hydrogen bonded regions indicating the dual modes remain in place, however ∼1/3 of the structure retains a B-DNA like structure and the non-canonical regions do not form more than 1one non-canonical hydrogen bond. Meanwhile the non-canonical stacking regions are more diffuse with less well-defined edges than in the implicit solvent case. However, looking at the average structures in Figure 2B and C we see interlinked structures similar to those in Figure 6, which suggests that while exact numerical values may differ, the overall structural response of the molecule is broadly similar in denatured areas. This is borne out further by the Curves+ analysis in Supplementary Figure S2 which shows very good agreement between the models, though the implicit solvent simulations are more variable in their average conformational parameters presumably due to an increased rate of exploration of configuration space, which would be expected for simulation in the absence of explicit water molecules.

**Figure 3.**
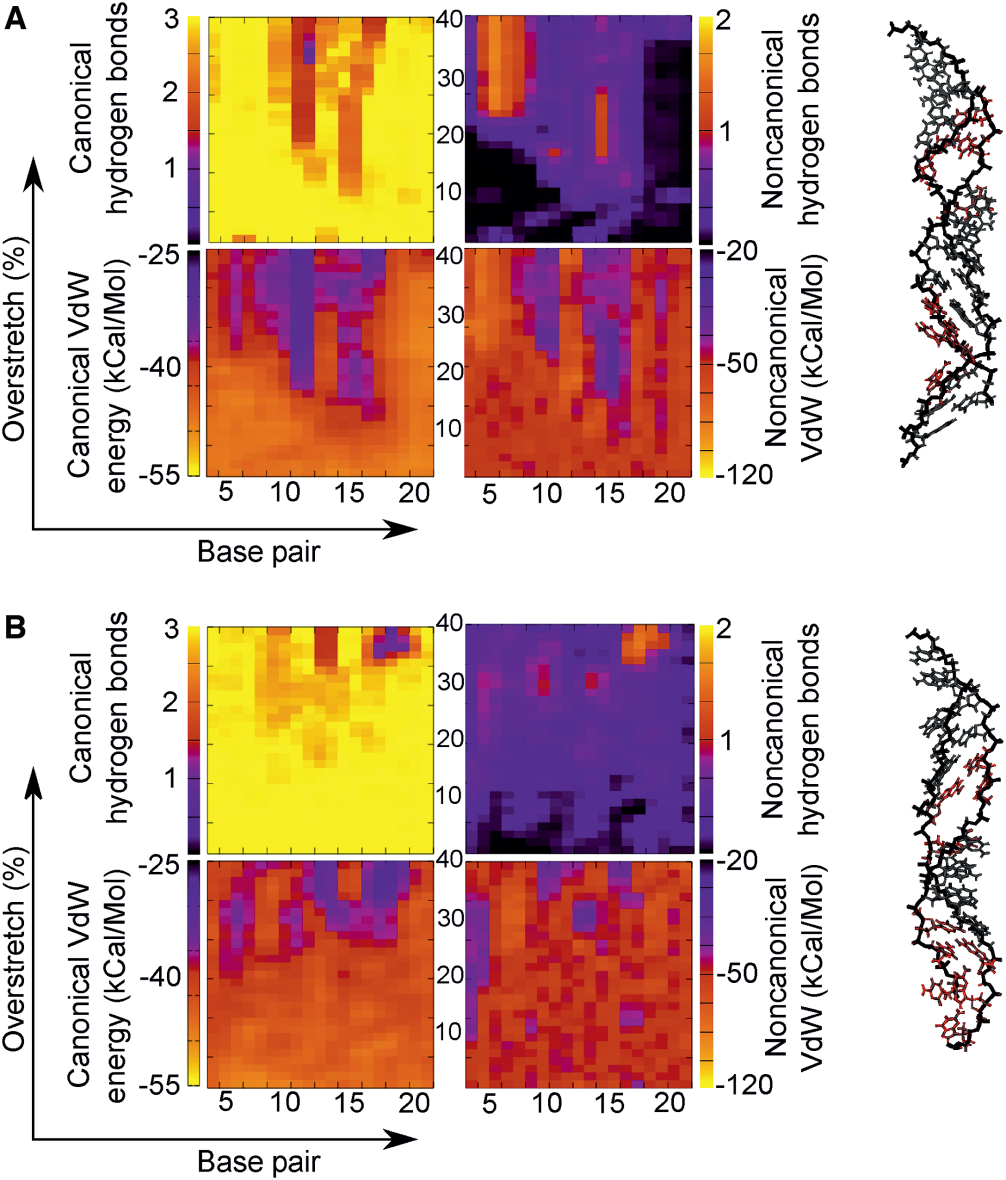
Hydrogen bonding and stacking for (CC)12 simulated in explicit solvent at 200 mM NaCl restrained at σ = 0.068 (**A**) and at σ = 0.068 (**B**). In each case, the structures are stable and show good correspondence with the implicit solvent simulation, with very little loss of canonical interactions. In the under-twisted case we see some increase of non-canonical hydrogen bonds but these do not correspond with a loss of canonical hydrogen bonds and hence are unlikely to be a conformational change. In the over-twisted case, the DNA remains virtually unchanged for the entire simulation in terms of base pair interactions. Red indicates a loss of >1 canonical hydrogen bond and grey indicates a relatively unchanged interaction landscape.

**Figure 4.**
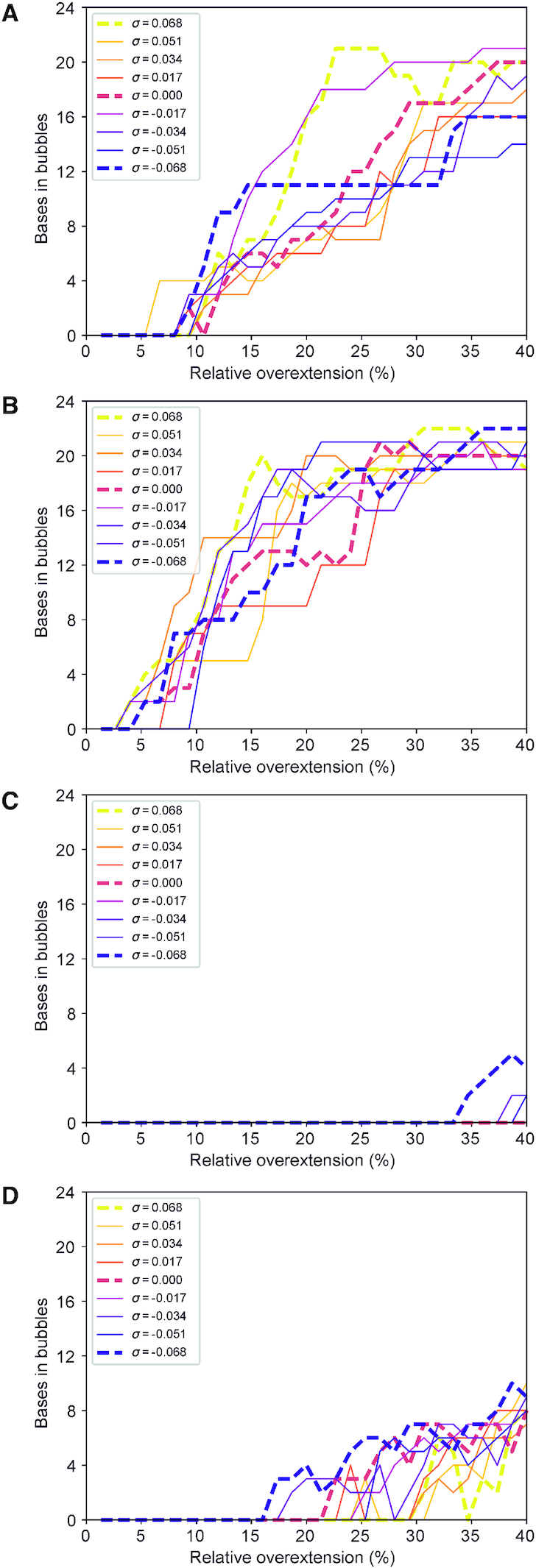
Number of bp involved in a melting bubble for each of the molecular dynamics simulations performed. (**A**) (AA)12 presents melting bubbles for under-twisted structures as early as 5% over-stretching, while over-twisted constructs form bubbles prior to ∼11% extension. (**B**) (AT)12 denatures in places almost immediately, for under-twisted structures at 2% over-extension in the earliest case, while surprisingly the last structure to form a melting bubble is under-twisted by σ = −0.051 and maintains its B-DNA structure until around 9% extension. (**C**) for (CC)12 melting bubbles are seen only for under-twisted structures, and those only late in the simulation compared to other sequences, with none produced before ∼33% extension. (**D**) (CG)12 is considerably more stable with under-twisted structures forming melting bubbles only after 15% extension and DNA with σ = 0.068 maintaining B-DNA hydrogen bonding up until 30% over-stretching.

For the (CC)_12_ explicit solvent simulations (Figure 3) we see a small deviation from the behaviour reported for implicit solvent. Specifically, in this case melting bubbles were formed for both systems, not only the under-twisted case as would be predicted from the implicit solvent melting bubble analysis as shown in Figure 4. However, we see melting bubble formation being initiated considerably earlier for the under-twisted case than the over-twisted, indicating that the broad observation from implicit solvent simulations—that melting is easier for negatively supercoiled systems—is correct. In both the explicit solvent simulations we see some increase in both non-canonical hydrogen bonding and stacking. Because of the lack of significant Watson–Crick hydrogen bond loss, this is likely due to the constrained geometry forcing bases close together rather than specific motif formation. Looking at the structures in Figure 3 it is clear that the explicitly solvated DNA preferentially creates localized ‘holes’ as opposed to broad-scale melting, while DNA which appears to be broadly B-like remains albeit with heavily inclines base pairs. Evidence of this is seen also in Figure 7B (top structure) although it is less striking.

Supplementary Figure S2 shows the Curves+ derived structural parameters for these simulations and their implicit solvent counterparts. It can be seen that there is an excellent level of agreement between the (CC)_12_ explicit and implicit solvent simulations and that the behaviour of the parameters is as would be expected, though later in the simulations fluctuations in average values between stretching events are likely due to the structure of the DNA breaking down and being incorrectly reported. In the worst case, the (AT)_12_, the deviation is below 10% and is likely due to incorrect readouts from non-canonical DNA. In general, all of the explicit solvent simulations here show good agreement with our implicit solvation simulations, and show that the results generated with the coarser method are reliable and a reasonable representation of the dynamics of the system. Comparison of the structural parameters with previous long time scale work (64,65) shows good agreement with published values of rise and twist, though with the twist parameter showing marginally more deviation. This is expected given that the DNA has been torsionally perturbed prior to the stretching simulations. Specifically, an average value of 3.42 Å in (64) for a GG base pair step is in excellent agreement with both explicit and implicit solvent simulations (Supplementary Figure S2). Average values of 3.42 Å and 3.31 for TA and AT steps are both in the range we report for the initial sampling windows for (AT)_12_ under both explicit and implicit solvation. As would be expected, we demonstrate twist values either above or below the reported 36.1° and 33.6° for CG and GC steps depending on the initial configuration of the molecule. Overall, the values are initially in accordance with reported values but exhibit some subsequent divergence following the applied mechanical perturbations.

### Sequence and stretch dominate over twist for the emergence of melting bubbles in both physiological and low salt conditions

To gain a general perspective of the degree of disruption of the B-DNA state among the whole set of simulations, we quantified the emergence of denaturation events. Figure 4 shows the effect of supercoiling and sequence on melting bubble formation through the quantification of the number of bp that have lost their canonical hydrogen-bond interactions for a particular stretching step (see Materials and Methods). Figure 4 shows the results for the 200 mM regime. We found that the results for the 50 mM simulations were highly comparable (Supplementary Figure S3). In each case, both AT-rich sequences present melting bubbles early in the stretching process (between 5 and 15% stretching in the 50 mM case and 5 and 10% in the 200 mM salt) that end up disrupting more than half of the DNA construct in the maximum extension. This behaviour was found to be generally shared on all supercoiling densities, suggesting a small dependence on torsional stress and a high level of fragility under stretching. Surprisingly, in low salt the (AA)_12_ simulation with σ = 0 demonstrated the largest melting bubbles, though it remains unclear if this was an ionic effect or due to a coarseness in the solvation model; this putative ionic strength dependence will motivate full characterization in future work.

In contrast, poly d(C)·poly d(G) is the most resistant sequence. In the low salt conditions, it does not form melting bubbles for any supercoiling density, although small melting bubbles are in evidence for negative supercoiling densities at extensions exceeding 34% in 200 mM salt. The strength of stacking interactions (66,67) and three hydrogen bonds between C and G are the probable reasons for preventing considerable perturbations. Poly d(C–G)·poly d(C–G) presents a significantly different behaviour compared to poly d(C)·poly d(G) as it forms melting bubbles on all the simulations affecting a minimum of 4 bp. It is also the sequence with the most supercoiling dependence: the earliest onset of a melting bubble is at an extension of ∼15% and occurs for σ = –0.068, while the last supercoiling density to create a melting bubble is σ = 0.068 at an extension of ∼30%, meaning that a changed supercoiling density allows the DNA to absorb twice as much extension before melting. This is in line with what could be expected—negative supercoiling tends to open the helix up and make it easier for base pairs to dissociate, whereas positive supercoiling is associated with packing the base pairs more tightly and constraining them in place (68). Significantly, there is only ever one melting bubble produced in the (CG)_12_ fragment, demonstrating that the perturbation is efficiently localized and grows in place rather than producing new bubbles (see further Results sections).

However, in these physiologically relevant supercoiling regimes even positively supercoiled DNA can form melting bubbles in a sequence- and extension-dependent manner. Thus, in general terms, our simulations show that the formation of denaturation bubbles is dependent on applied torsion only in a moderate way, suggesting that at these extensions the overstretching strain dominated over the stress due to twist. We obtain similar results for all ionic strengths, in contrast to previous experimental work on torsionally unconstrained DNA (6), although in our regimes the force-extension curves for DNA which cannot unpeel have been shown to be very similar (5). It is likely that implicit solvation is not yet detailed enough to state a conclusion with any certainty.

**Figure 5.**
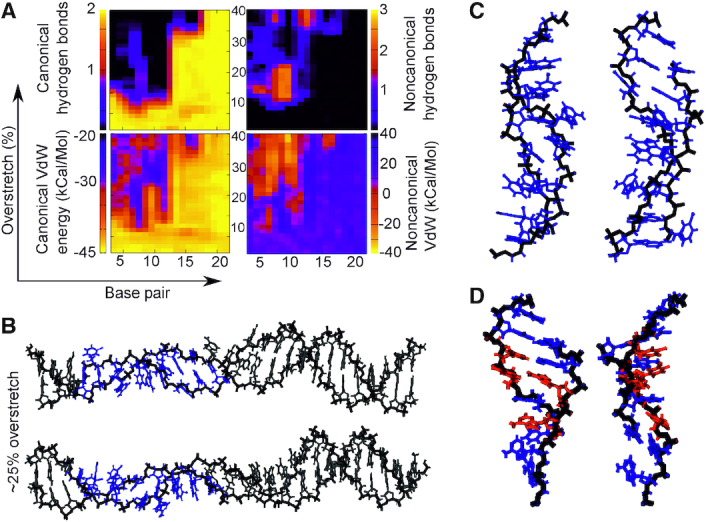
(**A**, **B**) Canonical/non-canonical hydrogen bonds and stacking energies (see Materials and Methods) as a function of applied over-stretch (up to 40%) on the simulation of (AA)12 at σ = −0.068 for 200 mM salt concentration. The insets show representative structures for an over-stretch of ∼24%. (**C**) detail taken of the structure in panel B. (**D**) detail from structures taken from the equivalent 50 mM salt simulation. Red colour indicates >2 non-canonical hydrogen bonds while blue indicates <2 non-canonical hydrogen bonds and <0 kCal/Mol non-canonical stacking energy.

**Figure 6.**
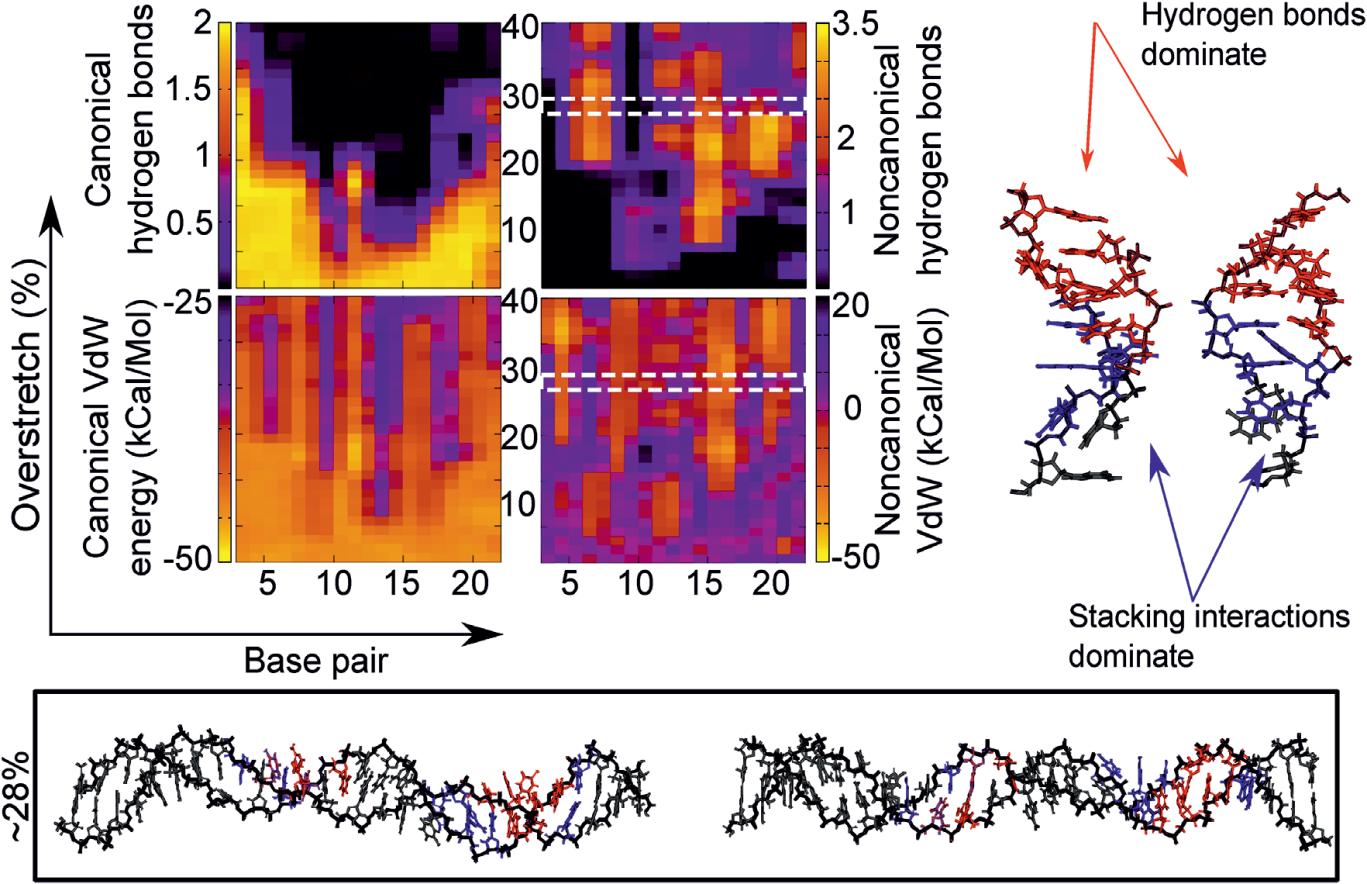
Canonical/non-canonical hydrogen bonds and stacking energies (see Materials and Methods) as a function of applied over-stretch (up to 40%) on the simulation of (AT)12 at σ = −0.068 in 200 mM salt. Insets show representative structures for an over-stretch ∼28%. Blue indicates <2 non-canonical hydrogen bonds while red indicates >2 non-canonical hydrogen bonds.

**Figure 7.**
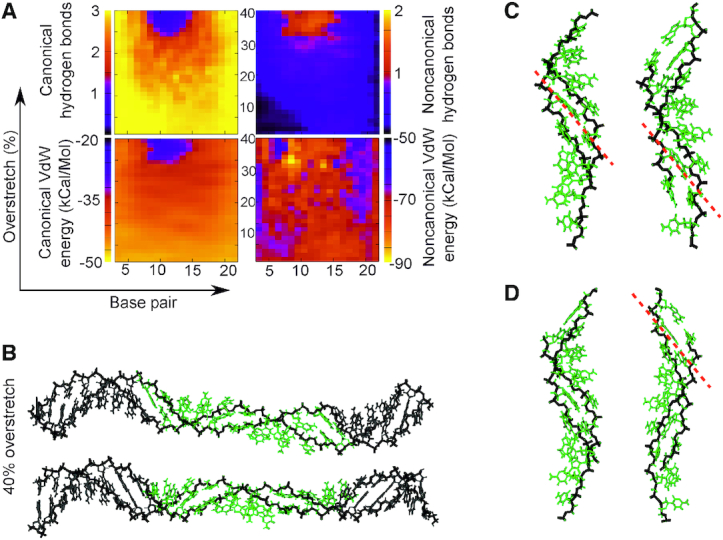
(**A**) Canonical/non-canonical hydrogen bonds and stacking energies (see Materials and Methods) as a function of applied over-stretch (up to 40%) on the simulation of poly d(C) ·poly d(G) at σ = −0.068 with 200 mM salt. (**B**) representative structures for an overstretch of 40%. (**C**) structures produced at 40% overstretch in 200 mM salt. (**D**) detail from the 50 mM salt structures also overstretched by 40%. Green indicates a loss of >1 canonical hydrogen bond. Grey indicates a structure close to canonical which has formed <1 non-canonical hydrogen bond and lost <1 canonical bond.

**Figure 8.**
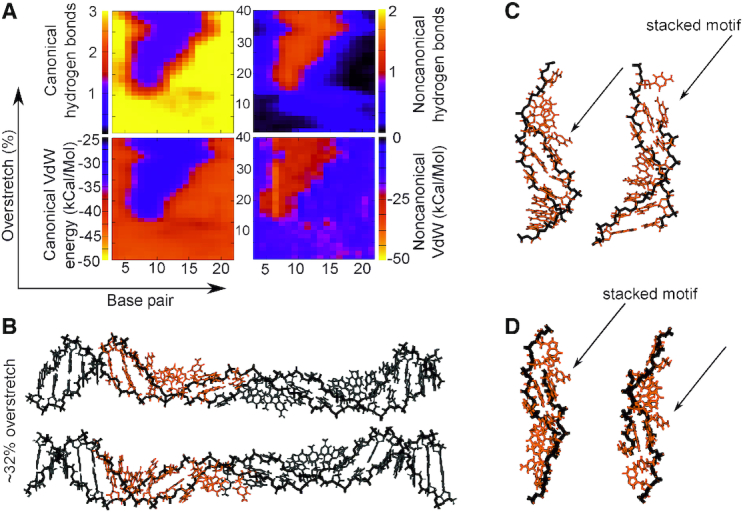
Canonical/non-canonical hydrogen bonds and stacking energies (see Materials and Methods) as a function of applied over-stretch (up to 40%) on the simulation of (CG)12 at σ = −0.068 in 200 mM salt. Two views of a structure from the simulation are shown in panel **B**. The inset panel **C** shows representative structures for an overstretch of 25 Å (∼32%) from the 200 mM salt simulations, while panel d) shows detail from the 50 mM structure. The orange colour scheme of the inset fragments indicates a structure stabilized by a broad coalition of all of the interaction types. Specifically, we see an absolute increase of ∼25 kcal/mol non-canonical stacking, a loss of two canonical hydrogen bonds, a gain of one non-canonical hydrogen bond, and an absolute decrease of around 20 kcal/mol for the canonical stacking energy.

In terms of sequence content, these results confirm the long-standing view that AT-rich sequences are more fragile compared with GC-rich sequences, although they also point to a far more complex picture than had previously been envisioned due primarily to the importance of stacking forces. Indeed, beyond melting bubble formation, the generation of stable structural motifs is entirely sequence specific (see the following Results sections), with the behaviour of poly d(A)·poly d(T) and poly d(C)·poly d(G) strikingly different from that of poly d(A–T)·poly d(A–T) and poly d(C–G)·poly d(C–G), respectively, indicating that simple approximations of DNA behaviour based on AT/GC content are reliable for comparatively long DNA sequences only in situations such as DNA melting for PCR considerations and single-molecule buckling point determinations (69) analysis. In the Results sections below Figures 5–8 show the outputs for each simulated DNA sequence at a level of under-twist comparable to that exhibited in live cells simulated in 200 mM salt concentration, to be contrasted against the equivalent over-twist simulations, and the equivalent low salt simulations, which we show in Supplementary Figures S4–S15. In the implicit solvation analysis, consistent colour schemes were used for each sequence across supercoiling densities and salt concentration. These are presented in Table 2.

**Table 2.** Colour schemes used for colouring the implicit solvation average structures

Sequence	Colour	Criterion
AA	Red	>2 non-canonical hydrogen bonds
AA	Blue	<2 non-canonical hydrogen bonds; <0 kcal/mol non-canonical stacking energy
AT	Red	>2 non-canonical hydrogen bonds
AT	Blue	<2 non-canonical hydrogen bonds
CC	Green	Loss of ≥1 canonical hydrogen bonds
CG	Yellow	Loss of 2 canonical hydrogen bonds, gain of 1 non-canonical hydrogen bonds, change in non-canonical stacking energy of ∼20 kcal/mol

### Poly d(A)·poly d(T) generates flexible dual motifs which may function as shock absorbers

Figure 5 and Supplementary Figure S5 shows the evolution of non-bonded interactions as the poly d(A)·poly d(T) ((AA)_12_) sequence is stretched at the negative supercoiled density of −0.068. We found that in both salt conditions canonical hydrogen bonds started to be significantly disrupted around a relative over-extension of ∼10%, with completely disrupted base pairs seen at 15% extension. Simultaneously, the disrupted WC hydrogen bonds begin to be substituted by non-canonical hydrogen bonds. These are short-lived in the 200 mM salt, lasting just until 22% extension, while in 50 mM salt only at around an extension of 35% do non-canonical hydrogen bonds largely disappear from the central region of the DNA. In both cases, this indicates a configuration in which a significant number of base pairs are involved in melting bubbles, though they persist at one end, likely due to edge effects from the immobilized base pair. As the WC hydrogen bonds are lost and non-canonical ones formed, stacking interactions follow suit. Canonical stacking energies reduce while non-canonical stacking increases, as may be expected for a DNA molecule whose bases are hydrogen bonding with unusual partners.

The structure extracted at the over-stretch step of 25% (Figure 5) shows detail of the denatured conformation, indicating two separate but linked modes of structural adaptation to the applied mechanical perturbation. In the high salt regime (panel C) we see that the dominant mode of stabilizing the duplex beyond ∼22% extension is non-canonical stacking, whereas in the low-salt we see both non-canonical stacking and hydrogen bonding. In each case, we see that the perturbed region suffers a collapsed backbone with corresponding rotation of the bases themselves outwards.

In the low salt regime, there is one region which contains a low number of non-canonical hydrogen bonds but which does exhibit significant non-canonical stacking. In the other region, there is the emergence of significant non-canonical hydrogen bonding and comparatively little non-canonical stacking. In this motif, the bases begin to form non-canonical hydrogen bonds with bases in the opposing strand adjacent to their natural WC pair. Thus, two separate motifs which rely on different stabilizing mechanisms are in evidence and initially coexist. However, in this case the stacking/hydrogen bond interplay does not persist beyond an extension of 35% in low salt and 22% in high salt. This is expected for the sequence with some of the weakest stacking interactions (see e.g. (66,67)) as well as fewest number of WC hydrogen bonds: the structure simply would not have sufficient stability to form a new, lower-energy structure, and thus melts, with that melting being affected by the electrostatic screening available from the surrounding solvent. For the trajectory containing this sequence at the highest level of over-twisting (σ = +0.068, see Supplementary Figures S4 and S6) we also observe denaturation bubbles. The structure is more stable in the early stages of overstretching as is expected from previous work (70). However, there are disruptions to the WC hydrogen bonding at ∼10% strain, and a large melting bubble quickly grows from the central region. Non-canonical hydrogen bonds are seen initially in the same areas, although once again these are lost as the stretching continues. Similarly, to the under-twisted case in Figure 5, our results suggest DNA would stabilize by engaging in new non-bonded interactions. With both the under-twisted and over-twisted structures forming collapsed-backbone structures, it seems that the early part of the torsionally constrained overstretching pathway is relatively insensitive to supercoiling density within the physiological range.

### Poly d(A–T)·poly d(A–T) generates stretch-resistant motifs stabilized by stacking and non-canonical hydrogen bonds insensitive of ambient salt concentration

The poly d(A–T)·poly d(A–T) ((AT)_12_) sequence showed a similar tendency to melt as the poly d(A)·poly d(T) under supercoiling density σ = −0.068 (see Figure 4), but the non-canonical interactions were found to be significantly more stable with applied extension (see Figure 6). As in the poly-d(A) fragment, non-canonical hydrogen bonds appear where canonical ones have broken, replacing the WC interactions entirely, and the stacking forces follow the same trend. At an extension of 10–15%, the DNA molecule forms a structure with a loose pattern of increasing and decreasing non-canonical hydrogen bonds, suggesting a new stable structural motif. Two distinct states are clearly seen in the structure. The first type of region is stabilized by non-canonical hydrogen bonds and canonical stacking (highlighted in red on Figure 6) and it has a B-DNA like macrostructure although the bases are slightly flipped away from the helical axis. The second type of structural motif is dominated by non-canonical stacking interactions, presenting low hydrogen bonding (highlighted in blue on Figure 6). The bases on opposite strands tend to be interlinked, suggestive of a conformation similar to zip-DNA (27). Our results suggest that this dual coexisting method of duplex stabilization is mechanically stronger than the previous structural motif emergent in poly-d(A) as it persisted until the end of the simulations. We observed the similar dual states for the rest of supercoiling densities in both salt regimes, although the pattern is less clear in over-twisted simulations with 50 mM (see Supplementary Figures S7–S9). For the simulation at σ = 0.068 and low salt regime, a significant central region of the construct maintains the WC pairing and canonical stacking of its B-DNA structure, but this is flanked by regions of increased non-canonical vdW energies and non-canonical hydrogen bonding (see Supplementary Figure S9). In the inset of Supplementary Figure S9, the dual stabilization modes can be clearly seen as in Figure 6 though they are established considerably later, at approximately 35% extension. Unlike poly-d(A), there is a melting bubble initiated at ∼20% strain which persists until the end of the simulations (see left hand inset in Supplementary Figure S9) in which there is stabilization only through the non-canonical stacking. Our simulations suggest that the observed stabilization modes are mostly invariable across supercoiling and ionic strength, with a caveat of some deviation at 50 mM and highly over-twisted DNA. Whether this difference is also supercoiling-dependent is unclear, but it seems likely that it plays a role given that the motifs appear always in tandem in under-twisted fragments while the only single motif occurred in the over-twisted DNA (see Figure 6).

Once again, in the over-twisted simulation, there are individual bases seen flipped outwards as a consequence of a collapsing backbone, once again demonstrating that the pathway to a bases-exposed conformation without significant positive supercoiling exists early in the overstretching regime in AT rich DNA and that this is a pathway in which mechanical perturbation generally dominates over salt conditions.

### Poly d(C) ·poly d(G) has a high resistance to mechanical perturbation due to strong canonical stacking and hydrogen bonding

For both salt concentrations, poly d(C)·poly d(G) ((CC)_12_) fragment is the most stable of all the sequences. The under-twisted stretching simulation maintained most of the canonical hydrogen bonds and non-bonded interactions as seen in Figure 7 and Supplementary Figure S11. Base pairs adopt a steeply inclined configuration in a similar way to that previously described for S-DNA (2,8,10). We observed a slight increase in the non-canonical stacking interactions as the DNA is being pulled due to the more prominent role of the diagonal interactions (or cross-stacking), although these changes are much less dramatic and cannot be related to any other configuration apart from S-DNA. Similar S-DNA like configurations were observed on over-winding DNA (see Supplementary Figures S10 and S12 and inset structures), though there is a smaller level of structural alteration as opposed to under-twisted DNA. This indicates the tighter packing of highly twisted bases is a substantial factor in regards to determining the level of structural stability. The heatmaps look broadly similar between high and low salt conditions implying that the strength of the Watson-Crick hydrogen bonding is insensitive to local salt concentration at these levels, and that the stretching perturbations indeed dominate in this regime. In summary, it seems here that the canonical stacking and the three WC hydrogen bonds are sufficient to stably accommodate this level of perturbation.

### Poly d(C–G)·poly d(C–G) forms mechanically stable four bp motifs which may accommodate significant over-extension

We found that the behaviour of the poly d(C–G)·poly d(C–G) ((CG)_12_) sequence was significantly different from that observed for the poly d(C)·poly d(G) sequence, in spite of identical total CG content. The principal difference here is the presence of intra-strand stacking of RY and YR bases instead of YY and RR (Figure 8). At an extension of 14% we observed backbone collapse similar to that of the AT-rich sequence, with a resultant loss of around two of the canonical hydrogen bonds in favour of non-canonical bonds. As seen for other sequences, there is a corresponding increase in non-canonical stacking energy as canonical hydrogen bonds are lost. The structural details of Figure 8 indicate that some bases on opposing strands are interacting via inter-strand stacking instead of the WC hydrogen bonds, and these motifs are robustly generated in both salt concentrations examined here.

This interaction seems to facilitate the formation of a four nucleotide motif, which consists of two bases on one strand followed by two bases from the opposite strand and is similar to what have been described in previous studies (19,44). Our simulations indicate the emergence of a repeating higher order structure comprising two of these four nucleotide motifs in succession. This configuration is characterized by a high inclination of the bases almost perpendicular to their usual orientation and maintains the stacking order CGCG, which is maximally strong (66,67). The disruption here preserves the canonical structure in other parts of the fragment, and the novel conformation appears able to absorb significant additional perturbation, as it does not grow in size through the simulation. Interestingly, for over-twisted poly d(A).poly d(T) we see one half of this motif formed at a certain stretch (see Supplementary Figure S4), i.e. four stacked nucleotides, demonstrating that the specific perturbation plays a role in determining these motifs. However in the case for (AA)_12_ we do not see the repeat of the four nucleotide motif, and overall the four nucleotide motif appears to be a property only of the (CG)_12_ sequence, from the four sequence we employed.

For the over-twisted case (Supplementary Figure S13), the behaviour is qualitatively similar to that indicated in Figure 8 though with σ = 0.068 the structure is considerably more resilient to applied strain, with WC base pairing being lost and non-canonical interactions appearing after around 20% extension. As in Figure 8, the loss of WC hydrogen bonds and canonical VdW stacking correlates exactly with the rise in non-canonical hydrogen bonding and stacking. Once again, indicated in the inset in Supplementary Figure S13, the four nucleotide motif reminiscent of (19,44) clearly develops and is stable to the end of the simulation. Supplementary Figures S14 and S15 show the corresponding under- and over-twisted simulations in 50 mM effective salt, and once again the stable the four nucleotide motif is clearly in evidence.

That the four nucleotide motif is strongly conserved across salt concentrations and supercoiling densities is an important, unexpected result. While in other sequences the produced motifs were similar (see Figure 5 and Supplementary Figure S3, and Figure 6 and Supplementary Figure S4) in this case the motif produced is identical in every respect. Four bases tilt such that they are almost aligned with the helical axis, and stack around the outside of a collapsed backbone. These motifs appear separate and stable on their own. Perfect replication of this motif in the over- and under-twisted DNA suggests that there could be an important biological role for this motif either in stabilizing the DNA or in exposing the base sequence for replication, regulation, or repair, especially in mammalian promoter sites with significant CpG islands (43) and plant cell signalling pathways making use of a CGCG box (42). The motif we observe could be a mechanism for recognition regulation in cell in either of these cases.

## DISCUSSION

In this study, we have run an extensive set of molecular dynamics simulations deforming DNA within the biological regime of stretching and torsional forces for four different sequences in order to investigate the structural effects of the different type of perturbations and their sequence-dependence. The four DNA sequences chosen ((AA)_12_, (AT)_12_, (CC)_12_ and (CG)_12_) enable us to interrogate not only the impact of the overall GC/AT ratio but also the impact of the bp-order, which causes different strengths of stacking interactions, these being critical for DNA stability. In general, we find that the sequence is the main determining factor for DNA’s response to over-stretching, dominating over applied supercoiling density and ionic strength. We find that AT rich DNA is less stable under stretching than CG rich DNA, and find that both AT and CG repeating dinucleotide steps form unique structural motifs, while AA simply melts and CC retains a highly canonical structure. In both cases of motif formation, non-canonical hydrogen bonds and stacking interactions are stably formed.

Our simulations focus on the early-stage of the overstretching reaction (up to an over-extension of 40%), comparable to the range of DNA extension resulting from the activities of DNA-binding proteins, such as TBP and RecA (45). Likewise, torsional constraints correspond to supercoiled densities between −0.068 and 0.068, which are the values observed *in vivo* either for prokaryotes (39) or eukaryotes (38). In general terms, we observe that this level of mechanical stress disrupts the canonical B-DNA form in most of the cases. Numerous denaturation events occur, even for the most positively supercoiled DNA, suggesting a weaker influence of torsional stress compared with stretching perturbation, which seems to dominate in this physiologically relevant regime. The appearance of bases flipped away on over-twisted DNA is the first step for the formation of a P-DNA-like structure reported previously for extremely overstretched torsionally constrained DNA. Unlike previous work, however, we find novel sequence-dependent structural motifs along the transition pathway in the early stages of over-stretching which are stabilized by different combinations of mechanisms.

Overall, AT-rich sequences show higher propensity to melt compared with CG-rich sequences in agreement with previous studies (19,30,31,44), although striking differences are found whether sequences contain RY and YR intra-strand stacking or YY and RR.

Poly d(C)·poly d(G) appears to be significantly more resistant than the other G-rich sequence, poly d(C-G)·poly d(C-G), because the former is the sole sequence that does not form melting bubbles for many supercoiling densities, and the latter presents denaturation for all torsional constraints. An important point to note is that poly d(C-G)·poly d(C-G) is the sequence that shows the highest sensitivity to changes of DNA twist, according to the wide range of observed extensions at which DNA starts to melt, being the first one at 15% for σ = -0.068 and the last at ∼30% for σ = 0.068. In contrast, for the two sequences with 100% AT content, the structure of the double helix is broken for all supercoiling densities at extensions as low as 15%, although the stability of the resultant conformation is notably different.

**Figure 9.**
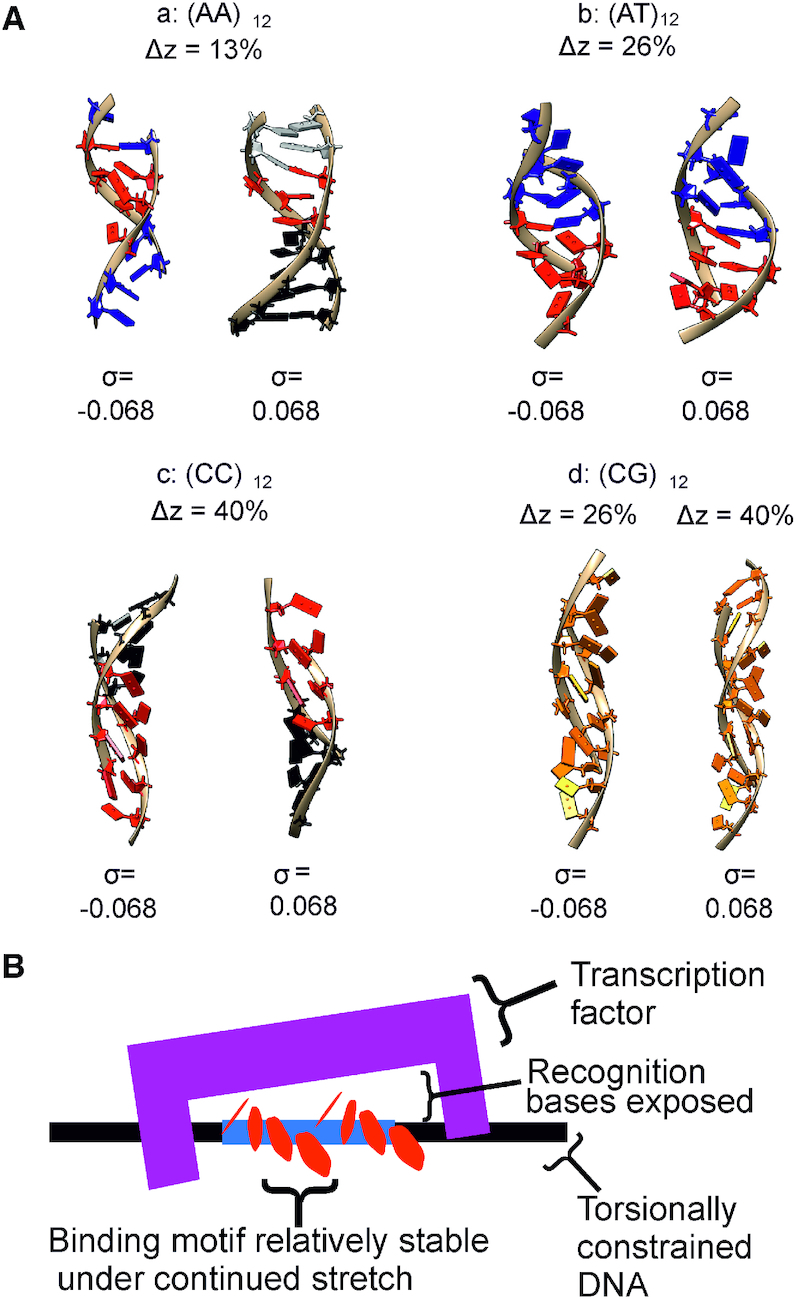
(**A**) Structural motifs seen in the four DNA sequences with σ = ±0.068 extracted from different over-stretching extensions from the 200 mM salt simulations. Blue indicates stabilization via non-canonical stacking while red indicates stabilization via non-canonical hydrogen bonding; the distinct interfaces between the two regions are therefore visible. For (CC)12 black is used because the structure is essentially intact. Colouring is taken from the appropriate Figures in the main text and Supplementary Information. (**B**) A cartoon schematic showing a possible interaction based on the motifs seen in the (AT)12 and (CG)12 simulations. Upon torsionally constrained overstretching, motifs appear which may then be recognized by transcription factors. Durability of the motifs upon additional over-stretching adds reliability to the interaction.

Figure 9A summarizes the key structural motifs that emerged as a result of the applied mechanical stress at 200 mM salt (equivalent 50 mM salt result shown in Supplementary Figure S16). At first glance, we can differentiate between GC-rich sequences, which develop a ladder-like geometry that can be classified under the umbrella of S-DNA, and AT-rich sequences, that present more apparent bubbles with bases flipped away. Nevertheless, DNA molecules with an alternating RY and YR sequence pattern produce alternative stable structural motifs due to the higher stability of non-canonical stacking interactions, which aligns well with previous work demonstrating pyrimidine/purine steps accommodating the majority of applied supercoiling density in a similar range to that used here (33) although that work was performed in the absence of a stretching load. Since both RY/YR sequences studied here have biological importance in promotion and regulation of genes (the TATA box (44), CGCG box (42) and CpG islands (43)) it is likely that the specific motifs we found have biological roles. Specifically, both the TATA and CGCG boxes require recognition from an associated transcription factor, while the CpG islands are found in ∼40% of promoter and exonic regions of mammalian genes. Mechanically stable structural motifs give a potential mechanism by which these regions may be activated or deactivated without significantly disrupting the local genome and without needing to tightly control supercoiling density which may be temporally or spatially varying (Figure 9B).

(AT)_12_ and (AA)_12_ polymers distort the helicoidal DNA shape with two alternating sequence motifs. The first one is similar in appearance to B-DNA although bases are slightly opened towards the major groove and stabilized by non-canonical hydrogen bonds. The second one makes a clear melting bubble having very few hydrogen bonds, although we did observe some non-canonical stacking interactions. The different degree of strength in stacking forces seems to be the cause of poly d(A–T)·poly d(A–T) having higher resilience compared to poly d(A)·poly d(T).

A strong inclination of bp with respect to the molecular axis is what characterizes the S-like configuration of the two sequences with high CG content, although the conformations are somewhat different. While poly d(C)·poly d(G) has a regular structure and keeps most of the canonical non-bonded interactions, poly d(C–G)·poly d(C–G) presents a non-canonical 4-base stacking motif at expense of breaking some of the canonical ones.

In most of the simulations presented here, the presence of torsional constraints has given rise to melting bubbles at extensions far below those reported previously for unconstrained DNA stretched for instance by pulling on opposite 5′ ends (29). This is due to the DNA being unable to overwind to reduce torsional stress as shown previously (26,46). As the DNA *in vivo* is torsionally constrained by being part of a large tract or attached to a histone, it is therefore likely that upon stretching by molecular machines melting bubbles are produced more readily in the cell than previously thought.

At first glance our results, which indicate that AT rich sequences are more labile than CG rich fragments, appear in conflict with previously published work (69) which found more melting in GC rich DNA constructs under mechanical manipulation. However, there is in fact no contradiction here. In the study (69) the authors concluded that the melting behaviour was as a result of *increased* stability in CG rich regions. In their experiments, DNA could relieve torsional stress either through melting or by forming plectonemes, and the localized melting seen in CG rich DNA was ascribed to AT rich regions melting in preference to CG rich regions forming a plectoneme which is a considerably more energetic process. As a result, the predictions in this paper—that AT rich DNA is less stable and melts more quickly under mechanical perturbations?—is borne out by the experimental literature. The fact that Zhang et al (5) found very similar force-extension behaviour for topologically closed DNA in 20 and 200 mM salt, which are consistent with our simulation findings at 50 and 200 mM salt, however we would need to perform further simulations and indeed a full salt titration in order to assess the full extent of this ionic strength dependence.

The observed structures are similar to the ones observed in previous studies (20,22,44,71) confirming the improvement on the implicit description of solvent (52,53,72). Moreover, previous QM/MM simulations demonstrated the validity of the AMBER forcefield for significantly perturbed DNA (29) which gives confidence in the reliability of the structures found here. We estimated the forces due to stretching and found them to be a typical level of a few tens of pN or less, in reasonable agreement with prior single-molecule experiments and previous simulations. However, we were unable to estimate the persistence length or other similar bulk measure of mechanical-material properties due to technical limitations. Specifically, the contour length of the sequence we employed of ∼8 nm is considerably below the anticipated nominal persistence length of 50 nm and therefore the usual worm-like chain approximation is a poor predictor of mechanical response due primarily to greater relative influence of finite volume effects which are not considered in standard worm-like chain model. In similar studies of DNA, the flexibility of the molecule is assessed by looking at the overall bending of the fragment, but this is not accessible to us due to the end base pair restraints. We can, however, note that the forces applied are physically reasonable in the context of being in a physiological range and broadly consistent with single-molecule experiments performed by other teams (e.g. (6)) with the proviso that there are expected differences in forces due to the differences in DNA fragment contour length.

Our simulations show that the overstretching behaviour not only depends on AT and GC content, but also on the nature of the different type of base pair step. Thus, extrapolating these results, we think that oversimplified models based only on AT/GC ratio for describing the DNA tendency to flex beyond the canonical B-form might only be valid for a rough and ambiguous picture. To fully understand molecular machines and the biomechanical function of promoter regions such as the TATA and CGCG boxes and CpG islands, it is vital to capture the behaviour of individual sequences.

## CONCLUSIONS

The ‘physics of life’ is characterized by complex and emergent features with typically substantive heterogeneity of molecular components in a system. The challenges of cutting through such heterogeneity, for example as exhibited in the dynamic structural states of DNA, can be best overcome by studying one molecule at a time (73), now feasible with studies *in vitro*, in live cells and in computational simulations (74). In this study, we have focused on a computational strategy involving molecular dynamics simulations on four distinctly different constructs of DNA in order to investigate the effect of sequence on the emergence of structural motifs in response to physiological levels of mechanical twist and stretch. We find evidence that sequence differences contribute significantly to the emergence, or not, of mechanically stable motifs. The presence of such motifs may potentially aid in recognition and transcription (75). For each different sequence simulated, even positively supercoiled DNA was found to form melting bubbles at the early stages of overstretching, indicating that, in the physiological regime of mechanical perturbation we used here, stretch is the dominant perturbation. We also find evidence that specific structural transitions occur in short *ca*. five bp regions, possible even in the absence of any topoisomerase activity. Our findings reinforce the importance of sequence to DNA topology, of particular relevance to sequence differences in gene promoter regions, in which mechanical perturbations have crucial roles. For future insights, we look towards exploring a fuller range of sequeneces in atomistic simulations, as well as extending simulations to longer length and time scale using coarse-grained adaptations; it will prove invaluable to validate these findings further experimentally, enabled both through the development of new *in vitro* correlative force transduction and single-molecule imaging technologies, such as combining optical and magnetic tweezers with super-resolution fluorescence imaging (76), but also in exploring methods of live cell imaging applied to investigating protein localization and interaction kinetics with DNA sequences and substructures of different topologies, such as those involved ahead of translocating DNA polymerases during replication (77,78), using super-resolved live cell tracking methods previously applied to proteins and lipids (79), for characterizing mobility and stoichiometry properties of clusters of single fluorescently labelled biomolecules (80) in both prokaryotic (81) and eukaroytic (82) cells.

## Supplementary Material

gkz1227_Supplemental_Files
