# Supplementary material for: The emergence of sequence-dependent structural motifs in stretched, torsionally constrained DNA
Source: Nucleic Acids Res. 2020 Jan 13;48(4):1748–63. doi: 10.1093/nar/gkz1227 (PMC7038985; doi:10.1093/nar/gkz1227)
Supplement: gkz1227_Supplemental_Files [file gkz1227_supplemental_files.zip › supplementary_information_v53.pdf]

The emergence of sequence-specific structural motifs in stretched, torsionally constained DNA

Jack W Shepherd[1], Robert J Greenall[1], Matt I J Probert[1], Agnes Noy[1*], Mark C Leake[1,2*]

[1]Department of Physics, University of York, York, YO10 5DD, UK
[2]Department of Biology, University of York, York, YO10 5NG, UK
*To whom correspondence should be addressed.
Email: mark.leake@york.ac.uk Tel: +44 (0)1904 322697 Fax: +44 (0)1904 322214
Correspondence may also be addressed to Agnes Noy. Email: agnes.noy@york.ac.uk

a

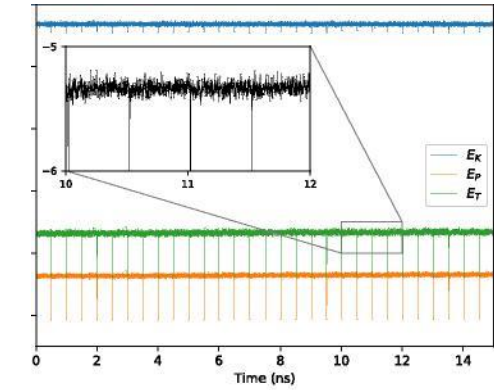

b

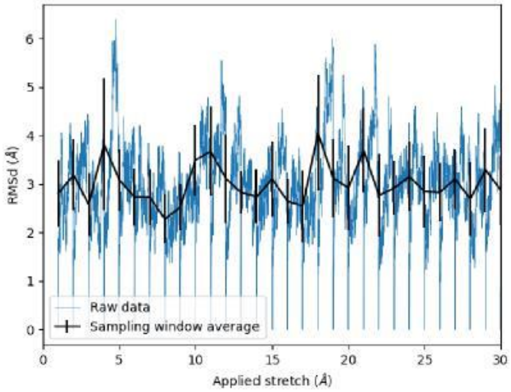

c

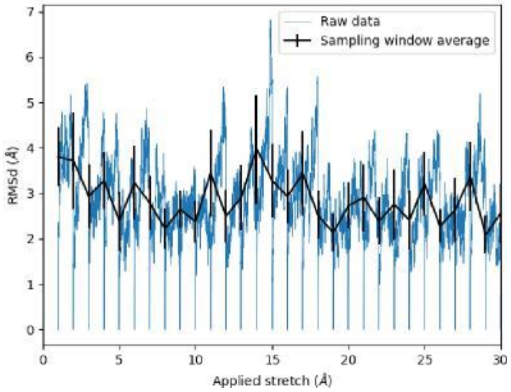

Supplementary Figure 1: a) Energy equilibration for (AA)12 over-twisted by σ =-0.068. The energies can be seen to be almost instantly equilibrated after a stretching event. b and c) RMSd from the first frame of each stretching window for (AA)12 with σ =-0.068 and σ =0.068 respectively. The range of RMSd is constant over the simulation and each sampling window indicating that the structures are in equilibrium.

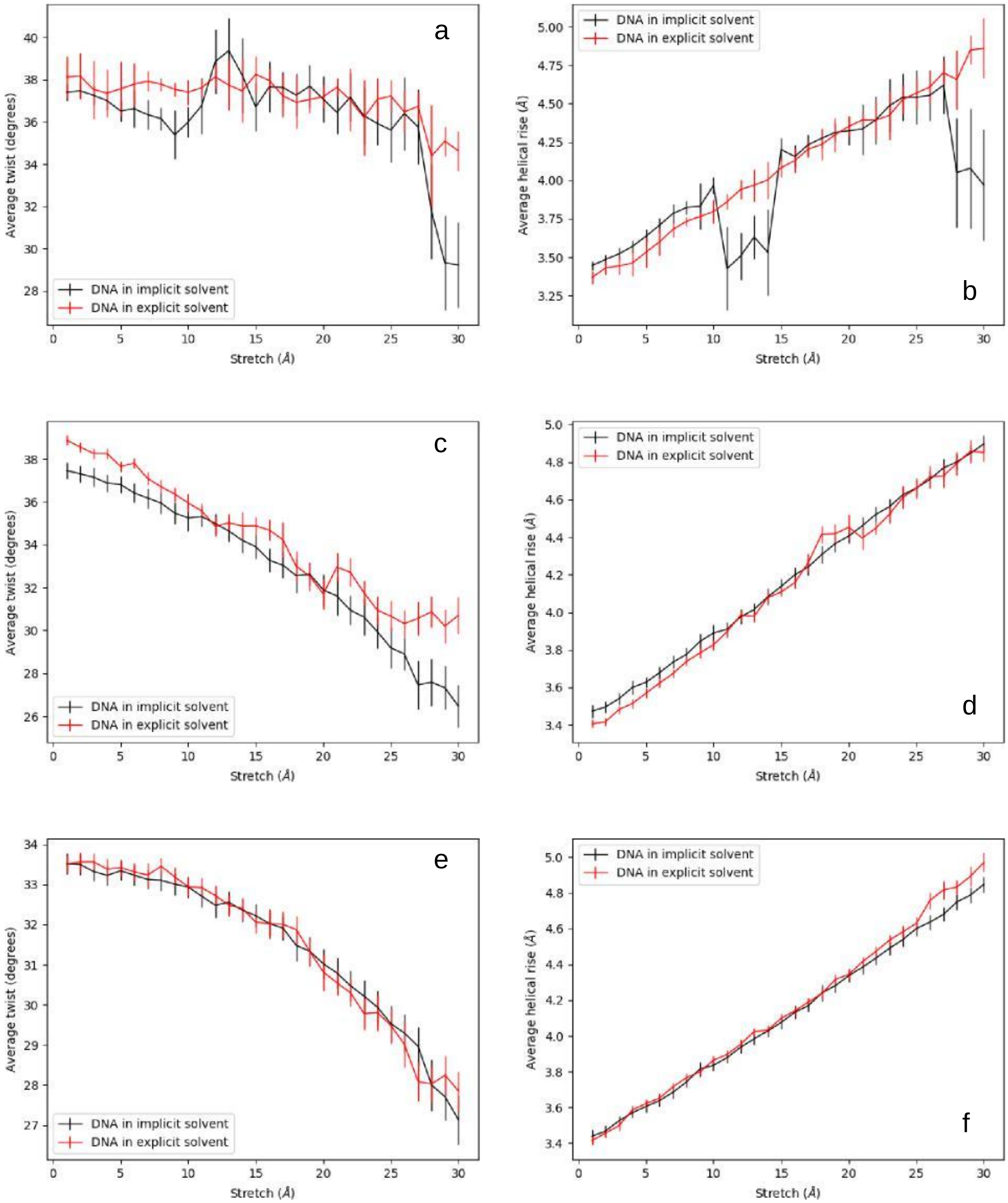

Supplementary Figure 2: Comparison of key structural parameters as calculated for simulations in implicit and explicit solvation models. a) Twist for $(AT)_{12}$ shows reasonable correspondence between the two. b) Helical rise for $(AT)_{12}$ is once again comparable. c) The twist comparison for $(CC)_{12}$ with $\sigma = 0.068$ is better than for $(AT)_{12}$ but not as good as the rise comparison in panel d) which is virtually identical. e) and f) twist and helical rise for $(CC)_{12}$ under-twisted to $\sigma = -0.068$ which shows excellent agreement. Each of these panels agrees well with previously published dinucleotide step parameters, allowing for variations due to torsional constraints and stretching.

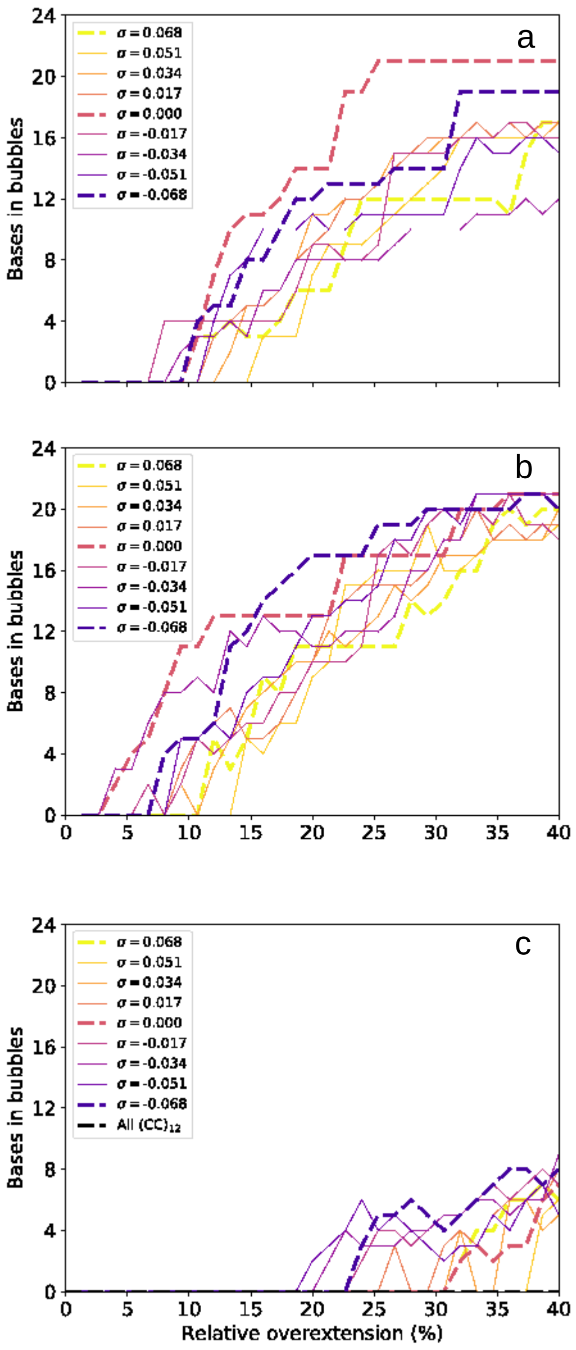

Supplementary Figure 3: Bubble formation for a) (AA)12, b)(AT)12, and c) (CC)12 and (CG)12 for simulations in implicit solvent with a salt concentration of 50 mM. (CC)12is by far the most stable, followed by (CG)12.

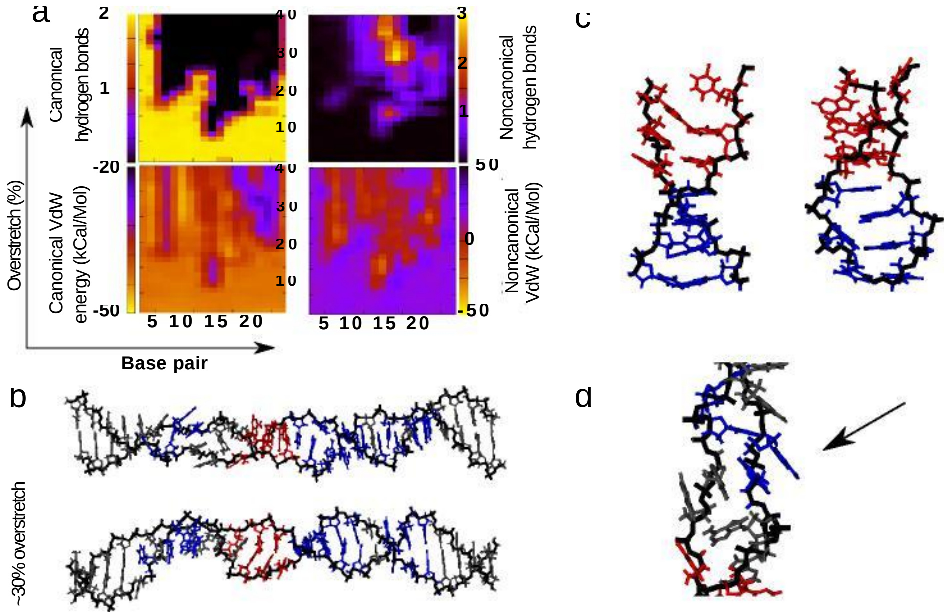

Supplementary Figure 4: Hydrogen bonding and stacking for $(AA)_{12}$ with σ =0.068 at effective 200 mM salt concentration. Panel c is a zoom of panel b, a structure taken from the umbrella sampling window with a 30% over-stretch. In panel d) which is another zoomed detail of panel b, the 4 nt (i.e. 2 bp) motif seen in all $(CG)_{12}$ simulations is in evidence, but is not repeated to form the full 4 bp structure. The grey nucleotides indicate that there is lower VdW stacking energy in that region, suggesting that this motif would be more unstable and may not survive contact with other biomolecules. Red colouring indicates >2 non-canonical hydrogen bonds per base pair and blue indicates non-canonical stacking energy <0 kCal/Mol. Grey indicates melted DNA with low stacking and hydrogen bonding.

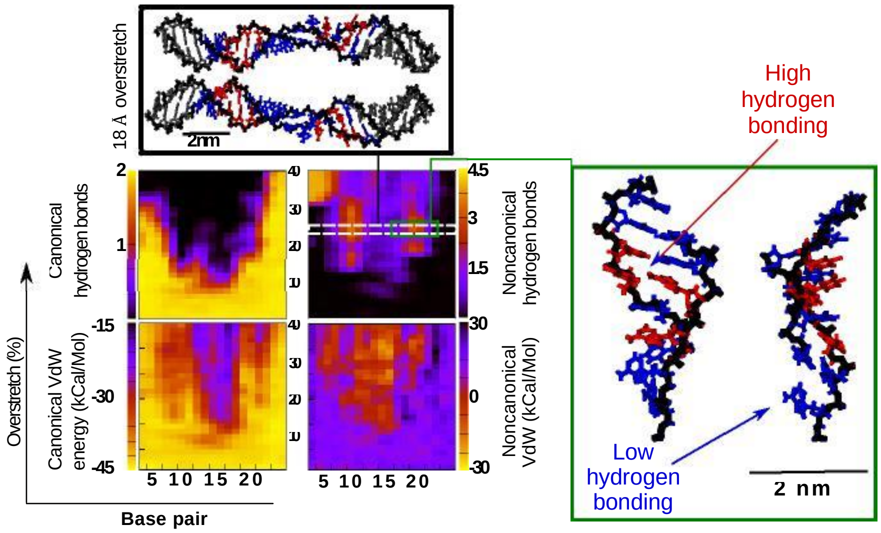

Supplementary Figure 5: Stacking and hydrogen bonding for $(AA)_{12}$ with σ =-0.068 simulated in 50 mM salt in implicit solvent. Red areas indicate high non-canonical hydrogen bonding (>2 non-canonical hydrogen bonds per base pair), while blue indicate high stacking (<0 kCal/Mol) – taken together, a dual motif is formed which co-exists in the middle part of the simulation.

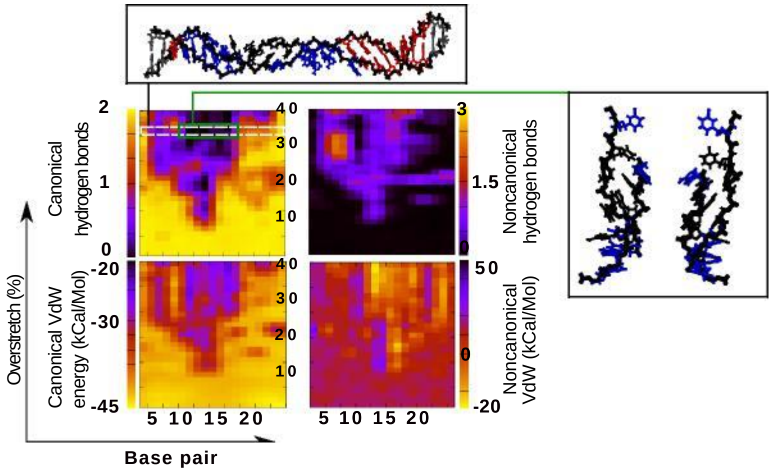

Supplementary Figure 6: Stacking and hydrogen bonding for (AA)12 with σ =0.068 simulated in 50 mM salt in implicit solvent. The dual motifs seen in Supplementary Figure 4 are not in evidence here, instead the duplex simply melts. Colouring as in Supplementary Figure 4.

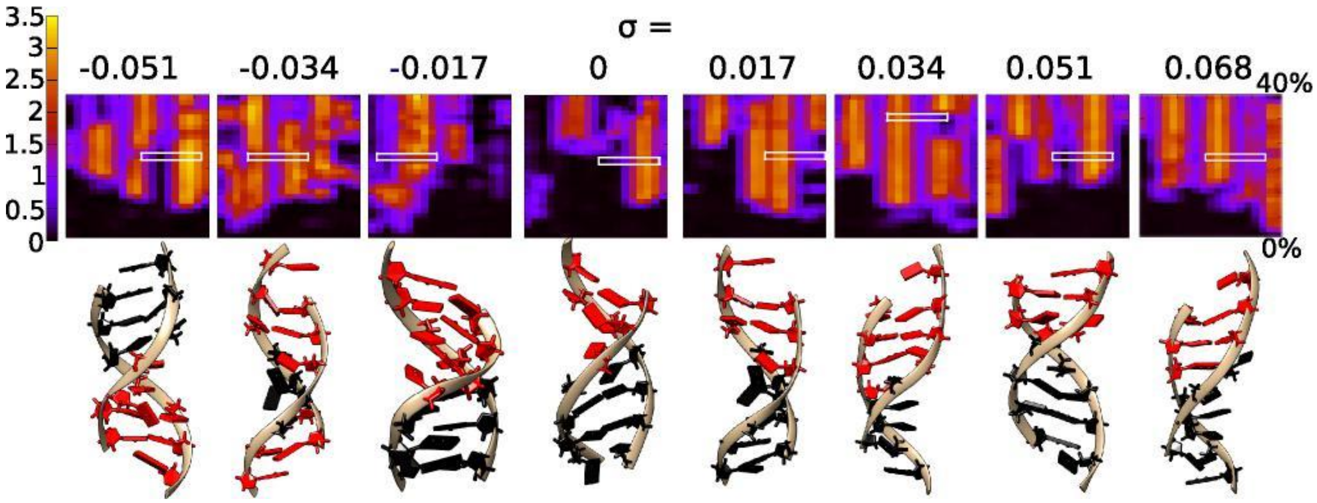

Supplementary Figure 7: Non-canonical hydrogen bonding for each of the implicit solvation (AT)12 simulations at 200 mM salt, along with representative average structures taken from the regions indicated by the white box. Red colouring indicate >2 non-canonical hydrogen bonds per base pair.

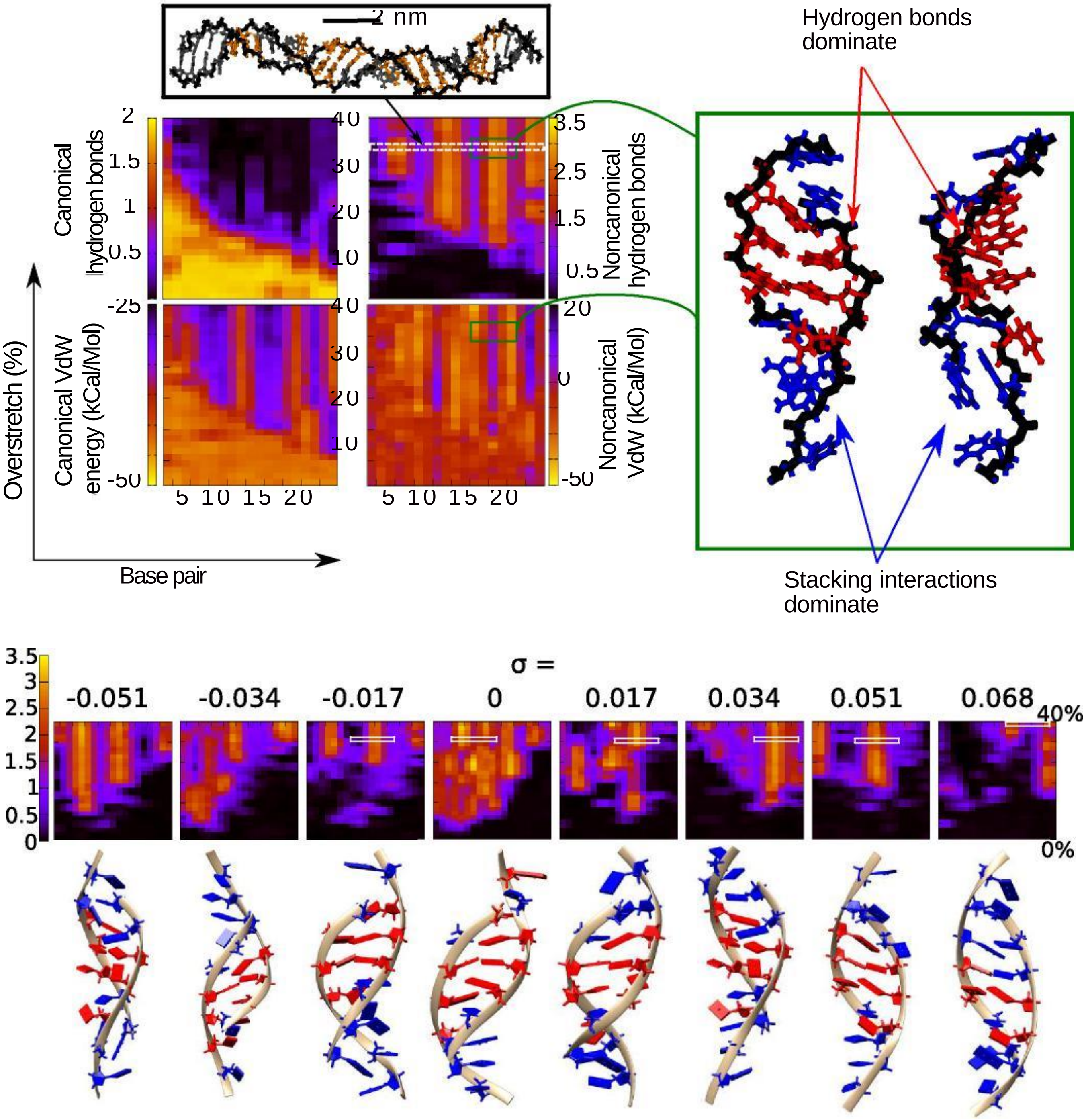

Supplementary Figure 8: Stacking and hydrogen bonding for (AT)12 simulated in 50 mM salt, and representative structures taken from ~35% over-stretch. Here are shown representative structures from other supercoiling density simulations, again at 50 mM salt. Colouring as in Supplementary Figure 6 with the exception that low non-canonical hydrogen bond regions are coloured blue rather than black.

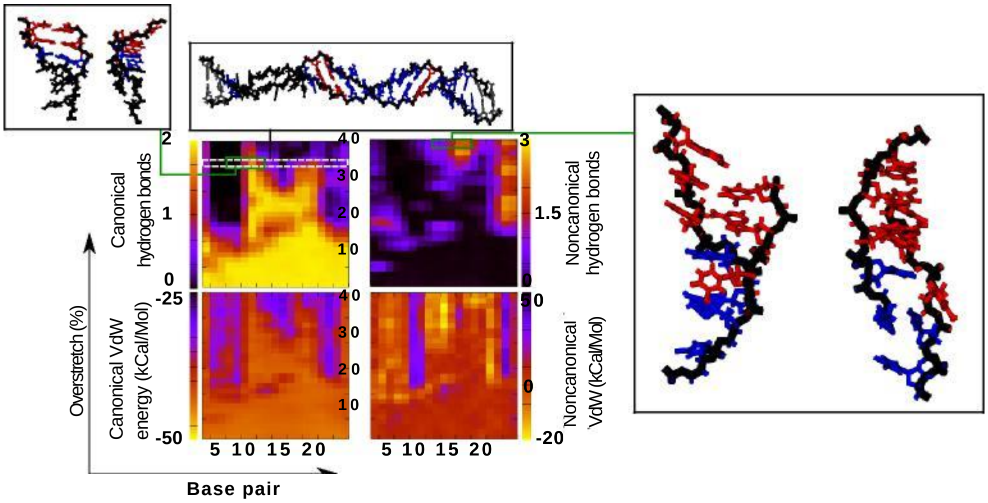

Supplementary Figure 9: Over-twisted (σ =0.068) $(AT)_{12}$ in 50 mM salt and representative structures thereof. The “striped” pattern seen in under-twisted and explicitly solvated $(AT)_{12}$ is much less clear indicating a supercoiling density role in the formation of these motifs. Colouring as in Supplementary Figure 4

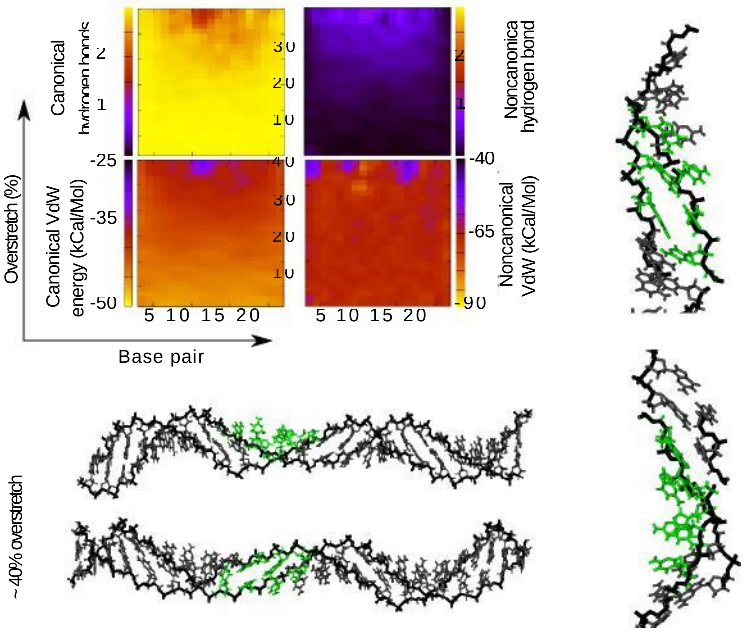

Supplementary Figure 10: Heatmaps for over-twisted (σ =0.068) simulations of $(CC)_{12}$ in 200 mM salt. Some canonical hydrogen bonds are lost (shown in green a loss of 1 canonical hydrogen bond) but overall the structure is very resilient. Average structure at 40% over-stretch. Note that we define melting bubbles to consist of a tract of two or more base pairs in which <1 canonical hydrogen bond remains on average. We therefore do not see melting bubble formation in this case as, although canonical hydrogen bonds are lost, around 2 remain for each base pair.

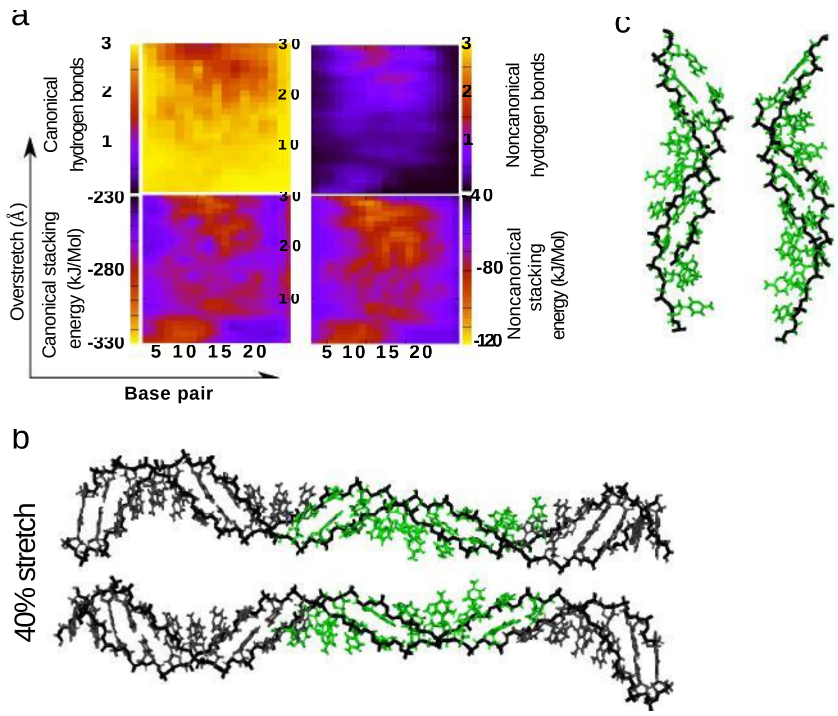

Supplementary Figure 11: Heatmaps and structures for (CC)12 under-twisted to σ =-0.068 in implicit solvent with 50 mM salt concentration. Once again the structure assumes a highly inclined conformation but does not melt. Colouring here is grey to indicate that the structure is largely unchanged and green showing a loss of >1 canonical hydrogen bond. The inset structures are taken from an extension of 40%, and panel c is a zoom-in of panel b.

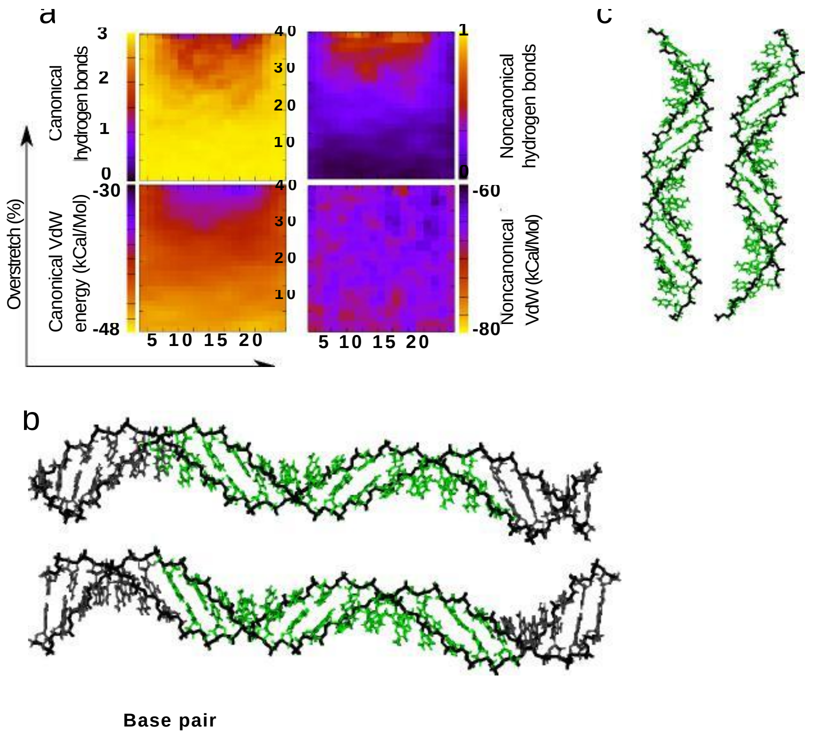

Supplementary Figure 12: Heatmaps and structures for (CC)12 over-twisted to σ =0.068 in implicit solvent with 50 mM salt concentration. The structure taken from an overextension of 40% (panel b) assumes a highly inclined conformation and loses ~1 canonical hydrogen bond, but overall remains relatively stable. Green indicates a loss of ~1 canonical hydrogen bond as in Supplementary Figure 9.

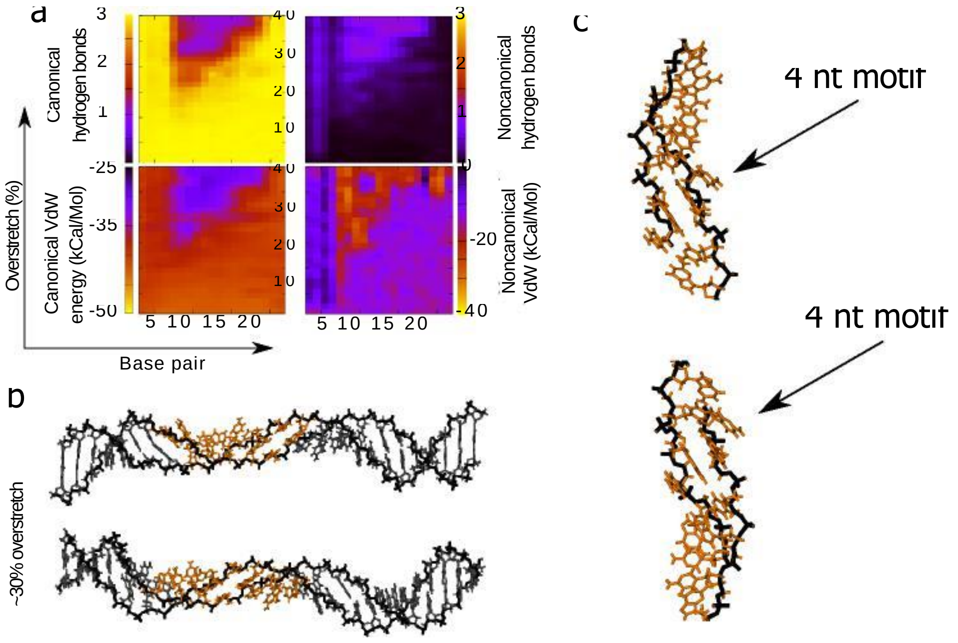

Supplementary Figure 13: Heatmaps and structures for (CG)12 over-twisted to σ =0.068 in implicit solvent with 200 mM salt concentration. As seen for the under-twisted structure, two 4 nt motifs are produced, creating one motif of the full 4 bp. These motifs are stable for much of the simulation and indicated in orange, characterized by a loss of 2 canonical hydrogen bonds, formation of around 1 non-canonical hydrogen bond on average and a sharp decrease in canonical stacking and increase in non-canonical stacking (> 20 kCal/Mol).

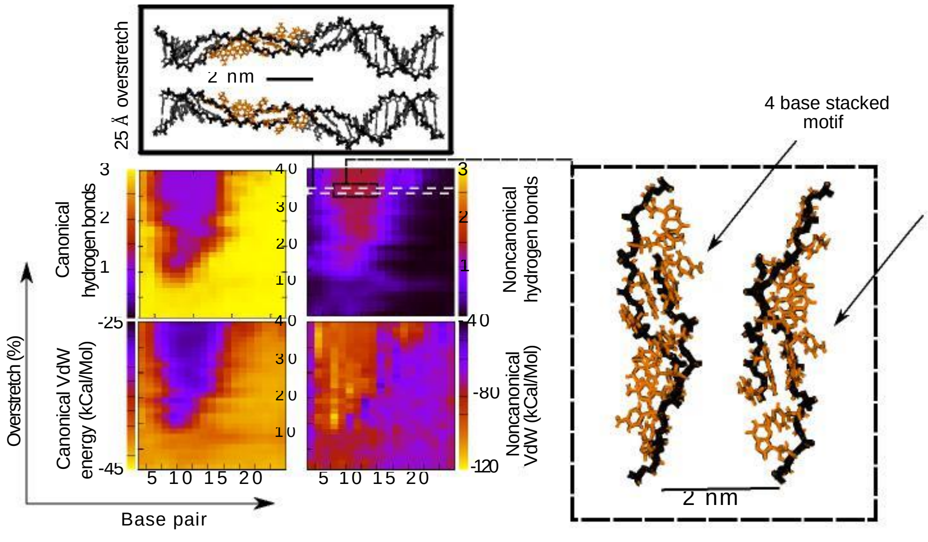

Supplementary Figure 14: (CG)12 simulated in 50 mM salt with σ =-0.068, showing again the 4bp motif formation. Colouring as in Supplementary Figure 12.

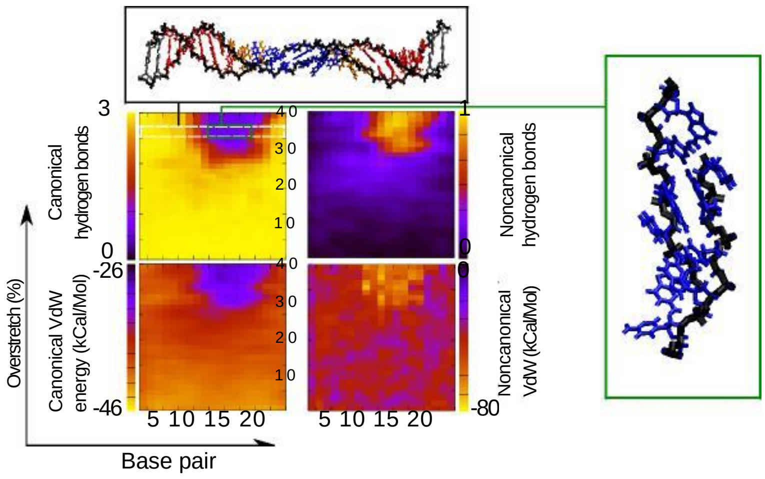

Supplementary Figure 15: (CG)12 in 50 mM implicit solvation with σ =0.068 once again forms the 4 bp motif. Colouring as in Supplementary Figure 12.

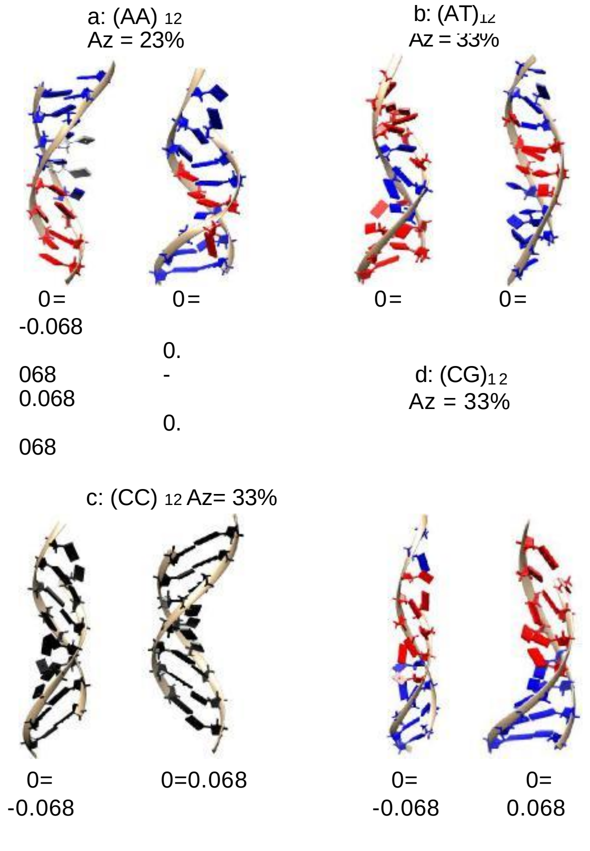

Supplementary Figure 16: Key features summarizing the structures seen in the 50 mM salt implicit solvent simulations. Red indicates high non-canonical hydrogen bonding while blue indicates high non-canonical stacking. Black in C) indicates the structure is essentially unchanged.

Supplementary Movie 1

Movie showing the trajectory for $(AA)_{12}$ simulated at a = −0.068 in implicit solvent at 200 mM effective salt concentration. Red bases are A and white bases are T.

Supplementary Movie 2

Movie showing the trajectory for $(AT)_{12}$ simulated at σ = −0.068 in implicit solvent at 200 mM effective salt concentration. Colouring as in Supplementary Movie 1.

Supplementary Movie 3

Movie showing the trajectory for $(CC)_{12}$ simulated at σ = −0.068 in implicit solvent at 200 mM effective salt concentration. Purple bases are G and green bases are C.

Supplementary Movie 4

Movie showing the trajectory for $(CG)_{12}$ simulated at σ = −0.068 in implicit solvent at 200 mM effective salt concentration. Colouring as Supplementary Movie 3

Supplementary Movie 5

Movie showing the trajectory for $(CC)_{12}$ simulated at σ = 0.068 in explicit (TIP3P) solvent with 200 mM NaCl concentration. Colouring as in Supplementary Movie 3

Supplementary Movie 6

Movie showing the trajectory for $(CC)_{12}$ simulated at σ = −0.068 in explicit (TIP3P) solvent with 200 mM NaCl concentration. Colouring as in Supplementary Movie 3

Supplementary Movie 7

Movie showing the trajectory for $(AA)_{12}$ simulated at σ = −0.068 in explicit (TIP3P) solvent with 200 mM NaCl concentration. Colouring as in Supplementary Movie 1.
